# Exploring the World of Double Nanohoops

**DOI:** 10.1002/anie.202516208

**Published:** 2025-12-26

**Authors:** Luisa Rzesny, Philipp Seitz, Birgit Esser

**Affiliations:** ^1^ Institute of Organic Chemistry II and Advanced Materials Ulm University Albert‐Einstein‐Allee 11 89081 Ulm Germany

**Keywords:** Conjoined bismacrocycles, Cycloparaphenylenes, Dimeric macrocycles, Dimeric nanohoops, Strained molecules

## Abstract

Conjugated nanohoops have become highly relevant compounds based on their unique conjugation, structural, optoelectronic, and morphological properties. Recent synthetic efforts have progressed to access more elaborate structures, incorporating two conjugated nanohoops covalently linked by a central linking unit, so‐called double nanohoops. Double nanohoops broaden the molecular diversity of strained nanocarbons, and they can show properties exceeding those of single nanohoops. They are often inherently chiral and attractive candidates as chiral‐polarized‐light emitters, and with two cavities are interesting supramolecular hosts. We herein provide an overview of double nanohoops reported to date, with loops built from only oligo(paraphenylene) units. We categorize their structures into phenylene‐linked double nanohoops, double nanohoops with X‐linkers, zig‐zag double nanohoops, lemniscular double nanohoops, and “other” double nanohoops. We identify four main synthetic pathways that are typically employed, making use of kinked precursors to oligo(paraphenylenes). We individually discuss properties and applications of each double nanohoop. This review shows that creativity and synthetic endurance can furnish unique double nanohoop structures with attractive chiroptical as well as supramolecular properties.

## Graphical abstract

As two conjugated nanohoops covalently linked by a central unit, double nanohoops are a relatively new class of strained nanocarbons with different properties to single nanohoops. This review gives an overview of all reported double nanohoops, categorizing their structures, syntheses, and discussing properties and applications. Creativity and synthetic endurance can reward double nanohoops with attractive chiroptical and supramolecular properties.

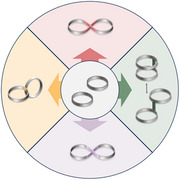

## 1 Introduction

**Figure 1:**
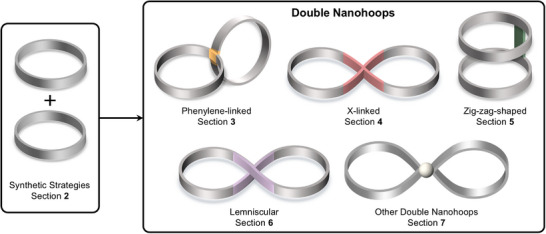
Categorization of double nanohoops discussed in this review by their geometry with corresponding text sections.

Curved conjugated hydrocarbons have fascinated chemists, physicists, theoreticians and materials scientists from their first proposition.^[^ ^1^ ^]^ The milestone development of synthetic routes to cycloparaphenylenes, starting in 2008,^[^ ^2^, ^3^, ^4^ ^]^ has enabled synthetic chemists to access conjugated nanohoops with a broad range of structural, optoelectronic and materials properties.^[^ ^5^, ^6^, ^7^, ^8^ ^]^ In continuing the quest to expand this family of strained conjugated nanohoops, a number of more complex molecular architectures, incorporating more than one nanohoop, have been reported in the past years. This includes, in particular, structures, in which two nanohoop loops are covalently linked by a central “linking unit”, which we herein call “double nanohoops”. These structures not only broaden the molecular diversity of strained nanocarbons, but they can also show interesting properties not known for “single nanohoop” molecules. Oftentimes, these double nanohoop architectures are inherently chiral, which—in connection with attractive luminescence properties—makes them potential candidates as chiral‐polarized‐light emitters. With two cavities they show rich supramolecular chemistry.^[^ ^9^, ^10^ ^]^ In this review, we provide an overview on all double nanohoops reported to date as well as their properties and applications. We discuss double nanohoops with loops built from only oligo(paraphenylene) units and categorize these by their geometry, which is determined by the type of linking unit chosen (Figure 1). The review starts with a section on synthetic strategies to double nanohoops, followed by sections on the individual types of geometries reported, namely phenylene‐linked double nanohoops, double nanohoops with X‐linkers, zig‐zag double nanohoops, lemniscular double nanohoops, and other double nanohoops.

As all of the discussed double nanohoops contain oligo(paraphenylene) linkers as loops, their optoelectronic properties are usually very similar to those of an [*n*]CPP of similar size and therefore not discussed in detail for every structure. We named the individual geometries of the double nanohoops by classifying the loops according to the number of *para*‐connected phenylene rings they are constituted of, and by naming and abbreviating the linking unit. Throughout all sections, we use the term “loop” to define structural parts that consist exclusively of *para*‐connected phenylenes and highlighted them in grey, while the linking units are highlighted in different colors, according to the corresponding sections. In contrast, the term “hoop” is used for the combination of a loop and (a part of) the linker, defining the shape of the macrocycle. Molecules with cylindrical architectures composed of two or more nanohoop segments are not considered double nanohoops within the scope of this review, since these systems lack a discrete linking unit between individual hoop motifs.^[^ ^11^, ^12^, ^13^, ^14^ ^]^ They are more appropriately classified as tubular or cylindrically shaped oligomers and are therefore not included here.

### 1.1 Synthetic Strategies to Double Nanohoops

**Scheme 1:**
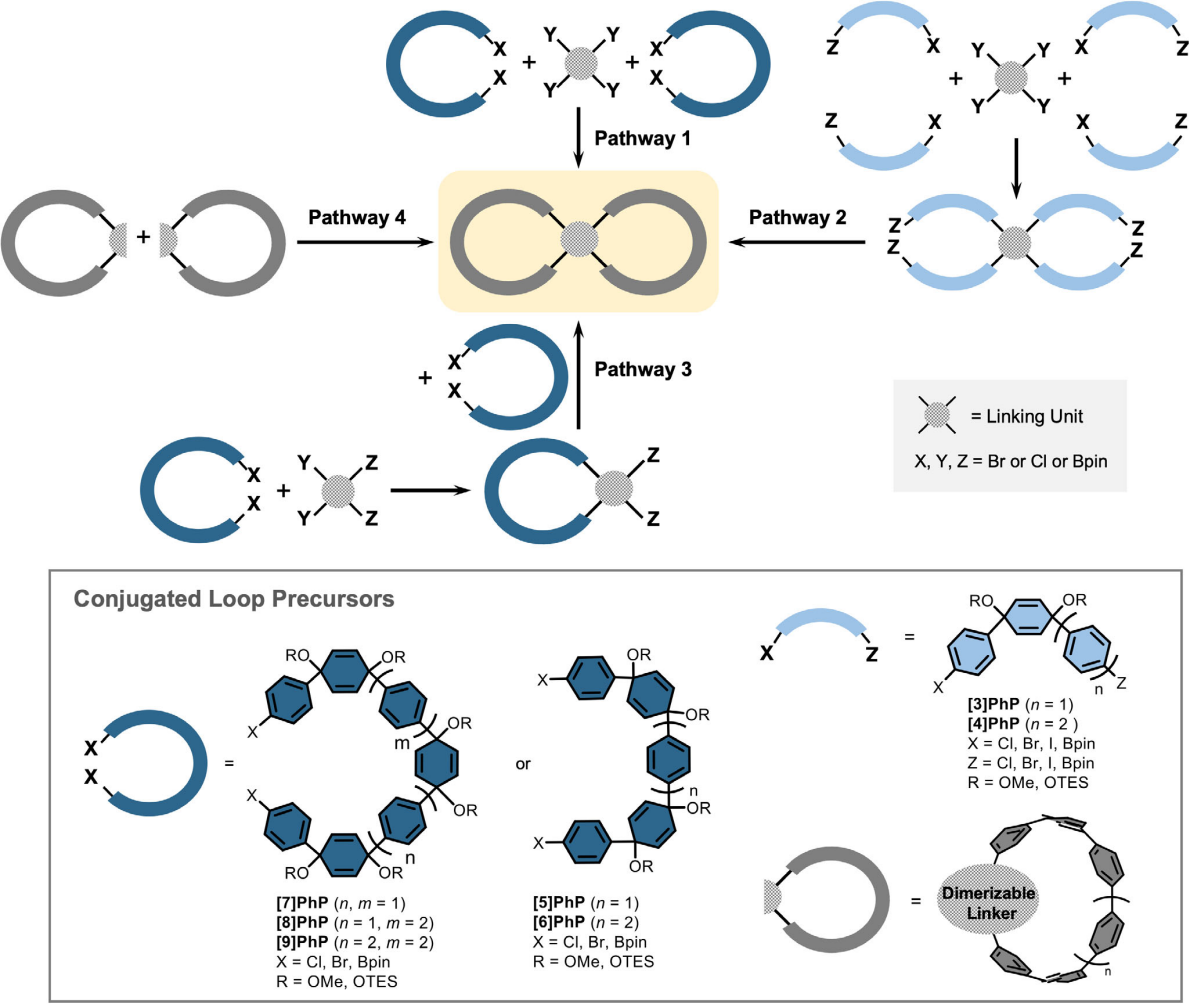
Schematic overview of the most commonly used synthetic pathways utilizing cyclohexadiene units in kinked oligo(paraphenylene) precursors as “conjugated loop precursors” of different sizes to obtain double nanohoops.

The synthesis of double nanohoops as architectures containing two covalently linked hoops presents significant challenges. These can be deduced to factors like structural instability, hoop strain, open‐shell character and low yields, in particular of cyclization and aromatization reactions. In the same way as for single nanohoops, bending the π‐system out of planarity in a strained macrocyclic architecture presents one of the major challenges. We identified four main pathways that have been used to synthesize double nanohoops (Scheme 1). They all employ kinked precursors to oligo(paraphenylene) units, a synthetic approach that has brought much success. It utilizes the unique geometry of 3,6‐*syn*‐dialkoxy‐1,4‐cyclo‐hexadiene units originally used in the preparation of the first cycloparaphenylenes (CPPs), and has been widely adopted.^[^ ^2^, ^15^ ^]^ These units are precursors to phenylenes, so‐called “masked phenylene units”, and provide access to relatively unstrained double nanohoop precursors by enabling macrocyclizations to occur. After successful macrocycle formation, these dialkoxy‐cyclohexadiene units can be aromatized by chemical reduction. Besides the synthetic strategy to bend the oligo(paraphenylenes) into strained loops, the geometry of the linking unit plays a critical role in double nanohoop design and also influences the choice of building blocks useable for nanohoop syntheses. Common examples are shown in Scheme 1.

Regarding the available precursors to oligo(paraphenylene) loops, components containing three to nine (masked) phenylene rings can be used, offering structural variety.^[^ ^3^, ^16^ ^]^ **[3]PhP**,^[^ ^2^, ^17^, ^18^, ^19^ ^]^ and **[4]PhP**,^[^ ^20^ ^]^ contain a single cyclohexadiene unit, introducing a bend of approximately 69° between the axes of the outer phenyl rings, while **[5]PhP**,^[^ ^21^, ^22^ ^]^ and **[6]PhP**,^[^ ^18^, ^19^, ^23^ ^]^ incorporate two such units, increasing the curvature. The largest known precursors, **[7]PhP**,^[^ ^17^, ^24^, ^25^ ^]^ **[8]PhP**,^[^ ^26^ ^]^ and **[9]PhP**,^[^ ^19^, ^22^, ^27^, ^28^ ^]^ feature three cyclohexadiene units, resulting in a “C”‐shaped structure, and are therefore frequently referred to as “C‐shaped precursors” in the literature. It should be noted that it is possible to prepare all said CPP precursors with triethylsilyl ether (OTES) or methoxy (OMe) groups for the alkoxy units OR. The reactivity is very similar, but the TES groups need to be deprotected prior to reductive aromatization to access the conjugated double nanohoop. However, the optimal choice heavily depends on the substrate. For intermolecular ring‐closing strategies, **[7]PhP**, **[8]PhP**, and **[9]PhP** are often the preferred choices, as their functionalized termini are optimally positioned to facilitate efficient cyclization with tetrafunctionalized small molecules, such as fourfold functionalized benzene derivatives (Pathway 1 in Scheme 1). Larger central linking units require larger precursors. For example, terphenyl‐like structures can only be closed by **[9]PhP** and usually not by **[7]PhP** using Pathway 1. A more favorable geometry of the coupling unit in Pathway 1, characterized by a kinked structure or a small distance between the ring‐closing positions, allows for the use of smaller and less kinked CPP precursors like **[5]PhP** or **[6]PhP**. Pathway 2 in Scheme 1 involves first attaching four kinked oligo(paraphenylene) precursors to the central linking unit, followed by intramolecular ring closure and aromatization. Using an asymmetric linking unit can enable the synthesis of double nanohoops with two different types of hoops (Pathway 3 in Scheme 1). Depending on the linking unit, this can result in two possible isomers. Sequential ring‐closing steps help control selectivity and reduce side‐product formation. This can be achieved either by using distinct functional groups for each cyclization step or by introducing the second set of reactive positions only after the completion of the first ring. In Pathway 4, a single nanohoop is first constructed, containing a “dimerizable linker”, and the double nanohoop is then synthesized from the single nanohoop using a suitable reaction, such as radical‐coupling, metal complexation, etc.

In all pathways, cross‐coupling reactions are employed to connect the linking unit to the kinked oligo(paraphenylene) precursors (Pathways 1, 3, and the initial step of Pathway 2). The most commonly applied one is the Suzuki–Miyaura reaction. For homocoupling reactions, which can be used in Pathway 2 for the intramolecular ring closures, two approaches have been established. The Yamamoto Cl/Cl or Br/Br homocoupling is generally preferred, as the corresponding halogenated precursors are typically readily available. However, if steric or geometric constraints prevent successful coupling, an oxidative boronic ester/acid homocoupling developed by Jasti *et al*. can be used. This is well‐suited to access strained systems, though it requires an additional synthetic step to prepare the boronic ester derivative from the halide.^[^ ^29^, ^30^, ^31^ ^]^ The functionalities X, Y, and Z in Scheme 1 therefore represent either chlorine, bromine, or boronic esters (typically the pinacol ester, Bpin), consistent with the coupling strategies described. Whether the boronic esters and halogen substituents for Suzuki–Miyaura reactions are localized on the central linking unit or the oligo(paraphenylene) precursors can vary depending on the specific case and on other factors, such as the difficulty of the boronic ester preparation or reaction yields. Both combinations are generally effective for successful coupling, and no clear preferred trend has been observed.

The last step of every double nanohoop synthesis is usually the reductive aromatization of the cyclohexadiene units, during which most of the hoop strain is built up. This is most commonly accomplished using SnCl_2_/HCl (*via* H_2_SnCl_4_)^[^ ^32^ ^]^ or lithium naphthalenide.^[^ ^2^ ^]^ It is worth mentioning that in highly strained hoops, side reactions, such as 1,3‐aryl shifts, can occur during this aromatization.^[^ ^33^ ^]^

### 1.2 Phenylene‐Linked Double Nanohoops

**Figure 2:**
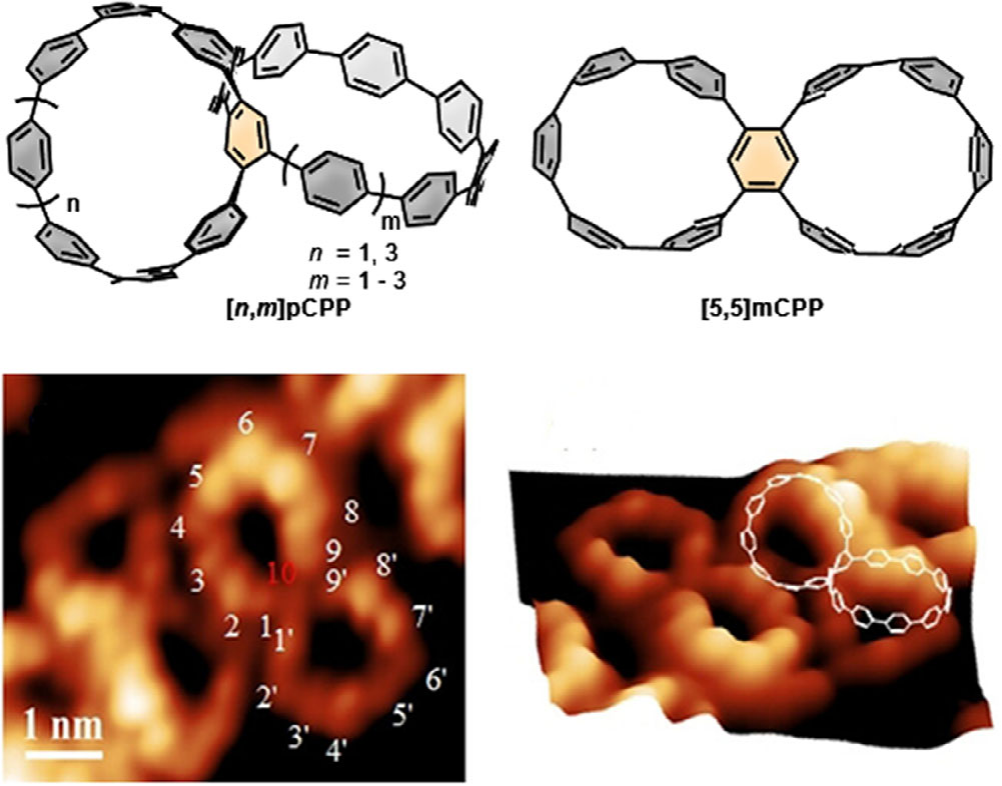
Phenylene‐linked double nanohoops **[*n*,*m*]pCPPs** and **[5,5]mCPP** with STM images of **[10,10]pCPP**. Reproduced from ref. [36] with permission from Wiley‐VCH GmbH. Copyright 2021 Wiley‐VCH GmbH.

As the first class of double nanohoops, we discuss those most closely related to [*n*]CPPs. They are solely composed of *para*‐connected phenylene units.^[^ ^33^ ^]^ Thus, these systems are the only ones in which the linking unit is included in the loop numbering. In this series, four different molecules were synthesized by the Du group, namely **[8,8]pCPP**,^[^ ^34^ ^]^ **[10,9]pCPP**,^[^ ^35^ ^]^ **[10,8]pCPP**,^[^ ^35^ ^]^ and **[10,10]pCPP**,^[^ ^36^ ^]^ (Figure 2) with optoelectronic properties listed in Table 1 in comparison to those of [*n*]CPPs.

**Table 1:** Optical properties of [*n*]CPPs^[^ ^37^ ^]^ and **[*n,m*]pCPP*s***.^[^ ^35^, ^36^ ^]^

Compound	*λ*_abs,max_ (nm)	*λ*_em,max_ (nm)	*Φ*_F_	Hoop strain (per Ph ring) (kcal mol^−1^)
[8]CPP	340	533	0.10	72 (9)
[9]CPP	340	494	0.38	66 (7)
[10]CPP	338	466	0.65	58 (6)
[8,8]pCPP	(346), 368	475	0.03	127 (8.5)
[10,8]pCPP	(340), 359	545^a)^	0.012	131 (7.7)
[10,9]pCPP	(340), 356	528^a)^	0.019	119 (6.6)
[10,10]pCPP	(340), 351	523^a)^	0.05	111 (5.8)

^a)^ Excitation‐wavelength‐dependent emission maxima.

The syntheses of these compounds followed Pathway 3 from Scheme 1, initially using a bis(benzyl ether)‐functionalized phenyl ring in the [10]CPP, serving as a linking unit, in which the benzyl ethers were subsequently converted into triflates. This bistriflate was then coupled with borylated **[7]PhP**, **[8]PhP,** and **[9]PhP** to produce the three distinct double nanohoops **[10,9]pCPP**,^[^ ^35^ ^]^ **[10,8]pCPP**,^[^ ^35^ ^]^ and **[10,10]pCPP**.^[^ ^36^ ^]^ The synthesis of **[8,8]pCPP** slightly differs. Here, 1,4‐dibromo‐2,5‐diiodobenzene was employed as the linking unit in two consecutive cross‐coupling reactions with **[7]PhP**.

The strain energies for these double nanohoops (in parentheses: per benzene ring) were calculated to be 111 kcal mol^−1^ (5.8 kcal mol^−1^) for **[10,10]pCPP**, 119 kcal mol^−1^ (6.6 kcal mol^−1^) for **[10,9]pCPP**, 131 kcal mol^−1^ (7.7 kcal mol^−1^) for **[10,8]pCPP** and 127 kcal mol^−1^ (8.5 kcal mol^−1^) for **[8,8]pCPP**. The hoop strain per benzene ring is in the same range as for [*n*]CPPs,^[^ ^37^ ^]^ but interestingly, the strain energies of the double nanohoops are slightly lower than for a single nanohoop of the same size. For example, the hoop strain per phenyl ring in **[8]CPP** is 9.0 kcal mol^−1^, whereas **[8,8]pCPP** exhibits a strain of 8.5 kcal mol^−1^ per phenyl ring. These double nanohoops **[*n*,*m*]pCPPs** show either a dual absorption or a very broad absorption. This was ascribed to a change in molecular orbital symmetry compared to those of [*n*]CPPs that leads to more complex electronic transitions.

Although these molecules are structurally very similar to their related CPPs, they exhibit novel optical properties. Their fluorescence quantum yields (*Φ*_F_) are significantly lower than those of the respective [*n*]CPPs (Table 1). For example, **[10,10]pCPP** has a *Φ*_F_ of 5%, while it is 65% for [10]CPP. The difference was explained by a high proportion of non‐radiative decay in the low‐energy emission. The most notable distinction in the optical properties is the excitation‐wavelength‐dependent emission observed in **[10,9]pCPP**, **[10,8]pCPP** and **[10,10]pCPP**. This behavior suggests anomalous anti‐Kasha excited‐state luminescence, where emission originates from different excited states, S_2_ and S_1_. This phenomenon can be attributed to the broken symmetry of these systems in comparison to [*n*]CPPs.^[^ ^38^ ^]^ **[8,8]pCPP** exhibits both aggregation‐caused quenching (ACQ) and aggregation‐induced emission (AIE) effects, leading to a significantly red‐shifted emission and near‐white‐light emission. The double nanohoops **[*n*,*m*]pCPPs** from Figure 2 are chiral, and for **[8,8]pCPP** the chiroptical properties are enhanced in the aggregated state, resulting in amplified circularly polarized luminescence (CPL) values. Consisting of two [10]CPPs connected through one phenylene unit, **[10,10]pCPP** can accommodate fullerenes, including PC_61_BM, within its cavity. Its hoop size closely matches that of [10]CPP, making it highly suitable for hosting C_60_ derivatives due to their nearly ideal geometric compatibility. Notably, this double nanohoop can even encapsulate two C_60_ derivatives (PC_61_BM), forming a peanut‐shaped 1:2 complex with binding constants of *K*_1_  =  7.5 × 10⁵ m ^−1^ and *K*_2_  =  5.9 × 10⁴ m ^−1^.

A fifth phenyl‐only double nanohoop, **[5,5]mCPP**, distinguishes itself from the previously described structures by the connectivity of the central linking unit.^[^ ^39^ ^]^ It consists of two [5]paraphenylene units bridged by a phenylene linking unit with two meta connections, making it structurally more similar to *meta*‐cycloparaphenylenes ([*n*]mCPPs). As a result, the optoelectronic properties of these systems more closely resemble those of [*n*]mCPPs rather than [*n*]CPPs. For instance, while [6]CPP exhibits no fluorescence, [6]mCPP displays a quantum yield of 22% with an emission maximum at 510 nm. Similarly, **[5,5]mCPP** shows a quantum yield of 25% with an emission maximum at 522 nm. These observations indicate that [*n*]CPPs are not an appropriate reference system for symmetry‐broken nanohoops.^[^ ^38^ ^]^ Due to the favorable geometry of the linking unit, the smaller CPP precursor **[5]PhP** (Scheme 1) was sufficient to achieve a two‐fold ring‐closing reaction with 1,5‐dibromo‐2,4‐diiodobenzene utilizing Pathway 1. Regardless of the tetrahalogenated linker used, only *meta‐meta* ring closing was observed, indicating that the geometry of **[5]PhP** exclusively aligns with this specific connectivity. Its crystal structure revealed that the two paraphenylene hoops are not perfectly coplanar with the plane of the linking unit, but are instead slightly bent above and below it, forming an angle of 30°. **[5,5]mCPP** is both a phenyl‐only double nanohoop and a double nanohoop with an X‐shaped linking unit, representing a perfect transition to the second class of double nanohoops discussed herein.

### 1.3 Double Nanohoops with X‐Linkers

**Figure 3:**
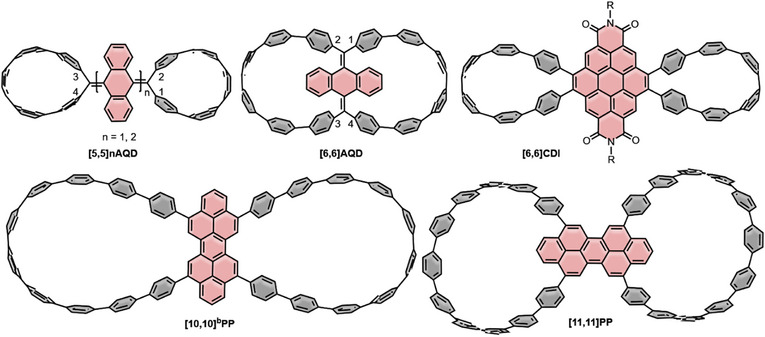
Double nanohoops with X‐linkers **[5,5]nAQD**, **[6,6]AQD**, **[6,6]‐CDI**, **[10,10]^b^PP**, and **[11,11]PP**.

As the second category we chose double nanohoops that contain X‐shaped central linking units (Figure 3) with optoelectronic properties listed in Table 2. This geometry is particularly advantageous for the hoop‐closing reactions, as the terminal positions of the linking units exhibit a kinked structure and therefore align well with the CPP precursor geometry.

**Table 2:** Optoelectronic properties of double nanohoops with X‐linkers.^[^ ^40^, ^41^, ^42^, ^43^, ^44^, ^45^ ^]^

Compound	*λ*_abs,max_ (nm)^a)^	*λ*_em,max_ (nm)^a)^	*ε* (10^4^ m ^−1^ cm^−1^)	*Φ*_F_ ^a)^	Hoop strain (kcal mol^−1^)
**[5,5]1AQD**	320, (440)	–	9.3	–	75.0
**[5,5]2AQD**	321, (440)	–	10	–	76.5
**[6,6]AQD**	320	478	10	0.204	49.4
**[10,10]** ^b^ **PP**	338^b)^	490	13	0.69	–
**[11,11]PP**	345, (434)	481	23	0.75	–
**[6,6]CDI**	348	532	–	0.011	–
**[6,6]AD**	336	494	18	0.59	–
**[6,6]PI**	331	464	23	0.63	–
**[9,9]THII**	335	464	15.9	0.80	82
**[11,9]THII**	334	456	16.2	0.80	76
**[11,11]THII**	335	437	16.8	0.95	70

^a)^ In CH_2_Cl_2._

^b)^ In CHCl_3_

The syntheses of **[5,5]nAQD** and **[6,6]AQD** (Figure 3), published by Sun *et al* ., demonstrate the effect of choosing different kinked oligo(paraphenylene) precursors for the hoop‐closing step.^[^ ^40^ ^]^ The synthesis starts with a tetrabrominated anthraquinodimethane (AQD) as a central linking unit for both species. Using Pathway 1 (Scheme 1) from reaction with **[5]PhP**, only **[5,5]1AQD** and [**5,5]2AQD**, connected via the 1,2‐ and the 2,4‐positions (see Figure 3 for labeling), were obtained. In contrast, following Pathway 2 exclusively yielded **[6,6]AQD**, where hoop closure occurred at the 1,4‐ and 2,3‐positions of the AQD linking unit (see Figure 3). Besides classical optoelectronic characterization, they were able to chemically oxidize **[6,6]AQD** with Magic Blue to the respective dication. X‐ray crystallography revealed a folded geometry for the neutral molecule and a doubly twisted geometry for the dication in which the linking unit is present as anthracene and not anthraquinodimethane. **[6,6]AQD^2+^** exhibits a linking number (*Lk*) of 2, as well as 48 π‐electrons, resulting in global Hückel antiaromaticity, which was further validated with 3D isochemical shielding surface (ICSS) calculations. Detailed investigations of the thermally accessible triplet state, ring rotation dynamics, absorption/emission behavior, and diradical properties of **[5,5]nAQD** were performed.

In 2024, the groups of Jasti and Xia published their work on the coronene diimide (CDI)‐based double nanohoop **[6,6]CDI**.^[^ ^41^ ^]^ By using synthetic Pathway 2 (Scheme 1), they obtained a donor‐acceptor (D‐A) structure that exhibits significant intra‐ and intermolecular charge‐transfer (CT) interactions and efficient spin‐orbit charge‐transfer intersystem crossing in the neat film. The rapid triplet formation was explained by the slipped herringbone packing (in contrast to CDI itself), which leads to cooperative intra‐ and intermolecular charge‐transfer interactions. Rapid generation of triplet states through intersystem crossings (ISC) in solution was revealed by transient absorption spectroscopy. This work demonstrates that integrating oligo(paraphenylene) hoops is an effective strategy for the development of triplet materials by impacting the crystal structure.

In 2021, the Juríček group reported on the two peropyrene (PP)‐based double nanohoops **[10,10]^b^PP**,^[^ ^42^ ^]^ and **[11,11]PP**.^[^ ^43^ ^]^ **[10,10]^b^PP** was synthesized *via* Pathway 1 using a tetra(biphenyl)‐extended PP linking unit that was synthesized by an oxidative dimerization of a functionalized phenalenyl radical, which yields a PP unit. The double nanohoop was fully characterized, including electrical conductivity measurements, and showed a 1:1 complexation of C_60_ in the solid state. Moreover, it was found that the crystallization conditions impacted the shape of the two hoops and could lead to an unprecedented lamellar packing in the solid state that enables their use in functional materials. The synthesis of **[11,11]PP** by the Juríček group utilizes a similar approach. Here, a phenalenyl radical‐containing nanohoop was first synthesized and then oxidatively dimerized to yield the double nanohoop **[11,11]PP** with the central PP unit in 32% yield, utilizing synthetic Pathway 4 (Scheme 1). This work presented one of the first dimerization approaches towards double nanohoops and demonstrated that CPP frameworks are appropriate platforms to control and investigate spin‐distribution and ‐delocalization through interaction of steric and electronic effects.

**Scheme 2:**
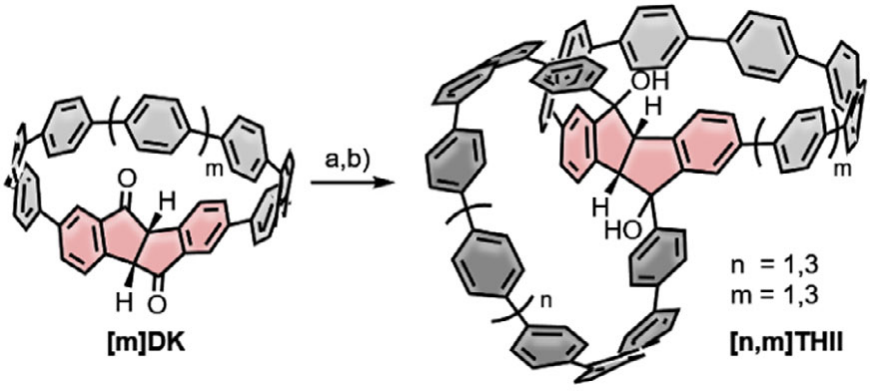
Synthetic approach toward tetrahydroindenoindene containing double nanohoops **[9,9]THII**, **[11,9]THII**, **[11,11]THII**. a) 1,4‐dibromobenzene, LiCl, Mg; b) Pd(OAc)_2_, SPhos, K_3_PO_4_; HCl, SnCl_2_.

In 2025, our group reported on the synthesis of a series of double nanohoops incorporating a tetrahydroindenoindene (**[n,m]THII**) serving as a twofold kinked linker, yielding **[9,9]THII**, **[11,9]THII**, and **[11,11]THII** (Scheme 2).^[^ ^46^ ^]^ The synthetic approach was based on a modified version of Pathway 3 (Scheme 1), wherein the monomeric diketo nanohoop **([m]DK)** was first functionalized using a Turbo‐Grignard reagent derived from 1,4‐dibromo‐benzene. This was followed by Suzuki–Miyaura cross‐coupling and subsequent reductive aromatization with H_2_SnCl_4_. The incorporation of the THII linking unit induced chirality, and the resulting enantiomers were successfully separated by high‐performance liquid chromatography (HPLC) using a chiral stationary phase.

Systematic analysis of the double nanohoops and the corresponding monomeric reference nanohoops revealed emergent properties, including altered photoluminescence quantum yields up to 95%, energy‐transfer dynamics, and increased asymmetry factors (*g*_abs_) values that exceed simple cumulative effects. These findings suggest an amplification of chiral properties due to the double nanohoop geometry. This enhancement is further reflected in the CPL values, although absolute CPL intensities remain relatively low across all compounds.

**Scheme 3:**
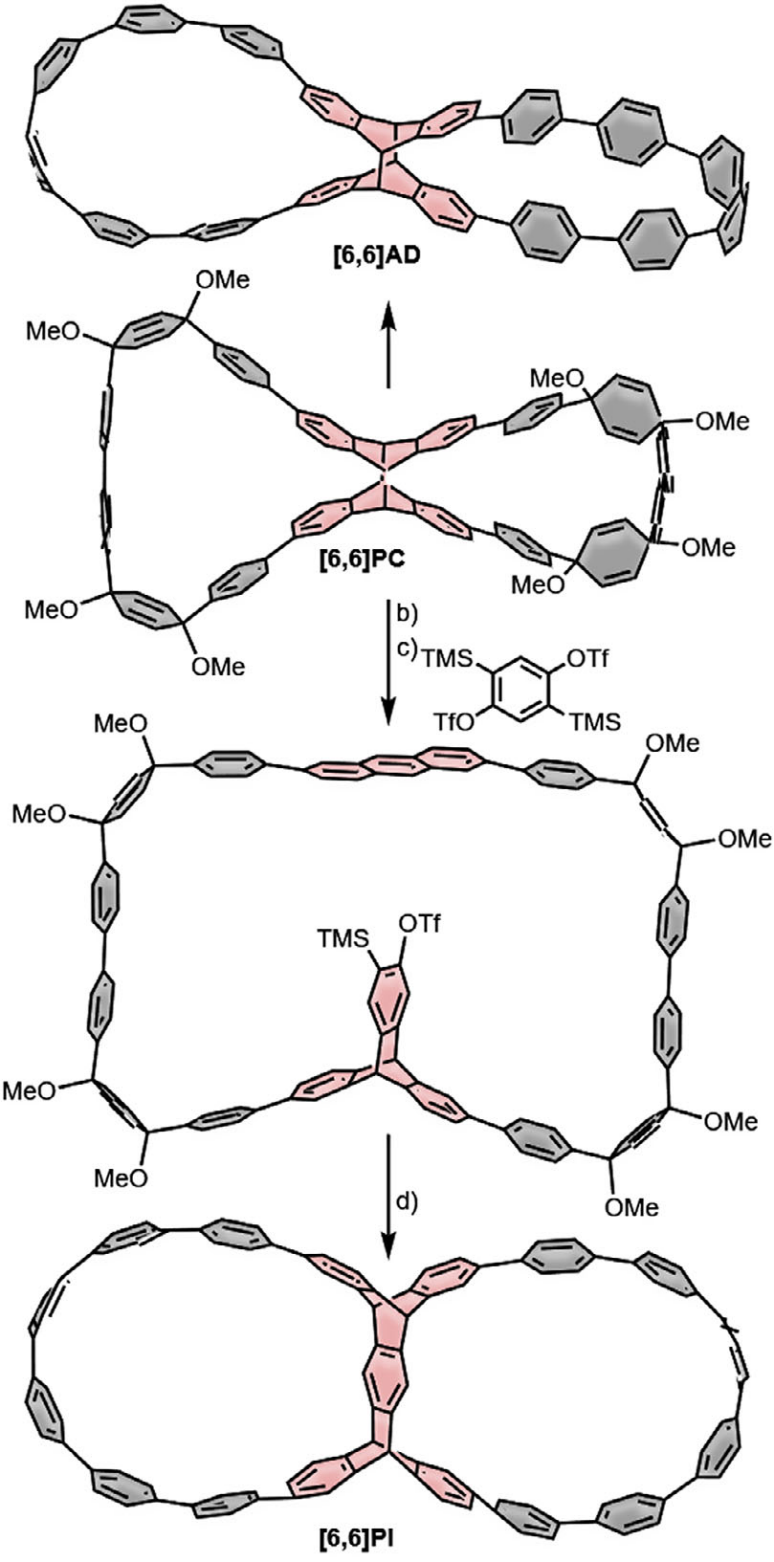
Synthesis of **[6,6]AD** and **[6,6]PI** starting from the same precursor. a) Sodium naphthalenide; b) 254 nm irradiation; c) CsF, 2,5‐bis(trimethylsilyl)‐1,4‐bistriflatebenzene; d) 1.) CsF; 2.) sodium naphthalenide.

In 2016, the Cong group reported the synthesis of double nanohoop **[6,6]AD** (Scheme 3).^[^ ^44^ ^]^ Interestingly, this double nanohoop could be converted into a single nanohoop through a retro‐[4+4]‐cycloreversion reaction of the central anthracene dimer unit. The X‐shaped linking unit was prepared by borylation of 2,6‐dibromoanthracene, followed by a [4+4]‐photodimerization, which yielded only a single stereoisomer. For nanohoop formation, Pathway 2 (Scheme 1) was selected with **[3]PhP** as kinked terphenylene precursor to achieve ring closure with an impressive yield of 95% using a Yamamoto homocoupling, resulting in **[6,6]PC** as intermediate. The double nanohoop **[6,6]AD** was thoroughly characterized, and a thermally induced cycloreversion at 175 °C successfully generated the bisanthracene single nanohoop in 72% yield.

By employing this strategy to post‐functionalize a macrocycle, they also synthesized compound **[6,6]PI**. The synthesis started with **[6,6]PC**, which underwent a light‐induced [4+4]‐cycloreversion to yield a bis‐anthracene macrocycle (step b) in Scheme 3. This intermediate was then transformed into the triptycene structure through a [4+2]‐cycloaddition with 2,5‑bis(trimethylsilyl)‐1,4‑bis‐triflatebenzene that was transferred to a benzyne with CsF (step c). The last two steps (d) were a second [4+2]‐cycloaddition, induced by benzyne formation with CsF in the triptycene unit, followed by reductive aromatization with lithium naphthalenide to yield the double nanohoop **[6,6]PI**. Due to the linking positions on the central pentiptycene linking unit, the resulting double nanohoop is chiral, and investigation of the chiroptical properties revealed *g*_lum_ values of up to 3.49 × 10^−3^.

### 1.4 Zig‐Zag Double Nanohoops

**Figure 4:**
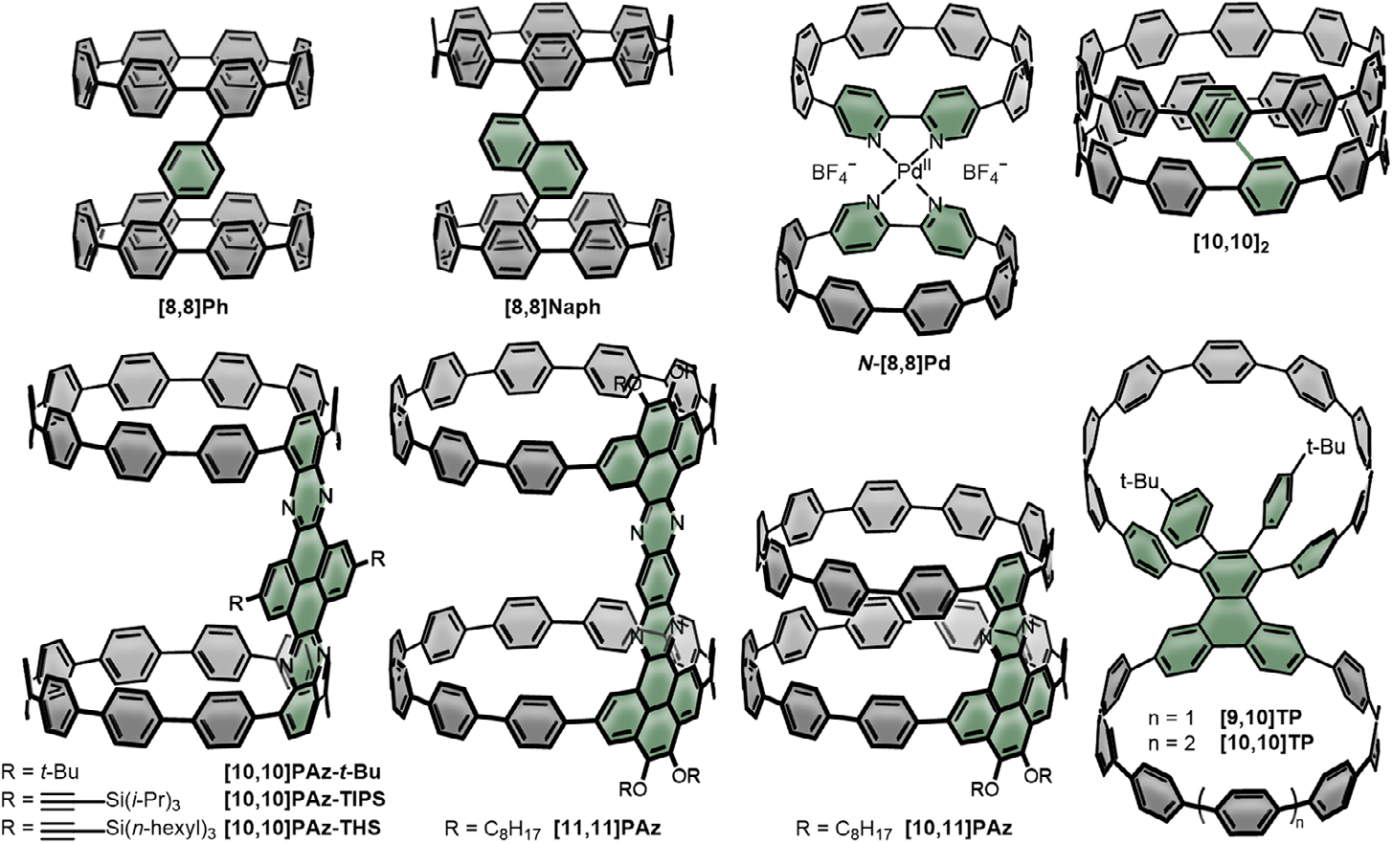
Zig‐zag double nanohoops **[8,8]Ph, [8,8]Naph, *N‐*[8,8]Pd, [10,10]_2_, [10,10]PAz‐*t*‐Bu, [10,10]PAz‐TIPS, [10,10]PAz‐THS, [11,11]PAz, [10,11]PAz, [9,10]TP, and [10,10]TP**.

As the third class of double nanohoops, we assembled the structures that, depending on the nature and geometry of the linker as well as the state of aggregation, can flip between a cis‐ and a trans‐conformation, and named them zig‐zag double nanohoops (Figure 4) with optoelectronic properties listed in Table 3. In the solid state, this behavior can lead to effective packing, while stable *cis*‐conformers in solution are interesting precursors for the synthesis of short nanotubes.

**Table 3:** Optoelectronic properties of zig‐zag double nanohoops.^[^ ^47^, ^48^, ^49^ ^]^

Compound	*λ*_abs,max_ (nm)^a)^	*ε* (10^4^ m ^−1^ cm^−1^)	*λ*_em,max_ (nm)^a)^	*Φ*_F_ ^a)^	Hoop strain (kcal mol^−1^)^b)^
**[8,8]Ph**	340	8.7	540	0.18	–
**[8,8]Naph**	340	17	540	0.15	–
**[10,10]PAz‐THS**	284, 340, 429	21.2	616	0.80	–
**[9,10]TP**	347	15.4		0.13	103.1
**[10,10]TP**	346	90.1		0.08	103.2

^a)^ In CH_2_Cl_2_.

^b)^ From homodesmotic reactions.

Jasti and coworkers presented two double nanohoops of this type, **[8,8]Ph** and **[8,8]Naph**, in 2012,^[^ ^47^ ^]^ four years after the first synthesis of carbon nanohoops from cyclohexadiene precursors.^[^ ^2^ ^]^ Similar to Pathway 4 in Scheme 1, but using non‐aromatized hoops, two non‐aromatized halogenated [8]CPPs were efficiently bridged with 1,4‐benzene‐ and 1,5‐naphthalene‐linkers through Pd‐catalyzed cross coupling (Figure 4).^[^ ^47^ ^]^ Compared to their parent molecule [8]CPP, both compounds showed similar optoelectronic properties, with a small deviance in the extinction coefficients and with increased *Φ*_F_ (Table 3), attributed to a more rigid structure. Cyclic voltammetry revealed (quasi‐)reversible oxidations of **[8,8]Ph** at *E*_1/2_  =  0.64 V *vs*. Fc/Fc^+^ and of **[8,8]Naph** at *E*_1/2_  =  0.68 V *vs*. Fc/Fc^+^. Computations showed that in solution and in the gas phase the *cis*‐conformation (as shown in Figure 4) is favored by 7 kcal mol^−1^ in **[8,8]Ph** and by 10 kcal mol^−1^ in **[8,8]Naph**, with a *cis*‐to‐*trans* isomerization barrier of 20 kcal mol^−1^. This stabilization was attributed to the increased van‐der‐Waals interactions between the macrocycles in the *cis*‐conformation. Both, **[8,8]Ph** and **[8,8]Naph**, as well as a theoretically modelled, directly linked [8]CPP dimer were proposed as CNT precursors due to their conformational preferences.

It was Itami and coworkers who published a larger version of the proposed dimer, **[10,10]_2_**, in 2014.^[^ ^50^ ^]^ Based on their strategy using cyclohexane derivatives as CPP precursors,^[^ ^51^ ^]^ and using a NaHSO_4_‐mediated aromatization, a monochlorinated [10]CPP was synthesized (Pathway 4 in Scheme 1) and dimerized in a Yamamoto homocoupling reaction. The structural aspects of monochlorinated [10]CPP and **[10,10]_2_** were compared by means of NMR spectroscopy and mass spectrometry. In DFT calculations, the *cis*‐ or closed conformation of **[10,10]_2_** (as shown in Figure 4) was revealed to be more stable (by 5.1 kcal mol^−1^) than the *trans*‐ or open form, and an isomerization barrier of Δ*G* ^‡^  =  8.9 kcal mol^−1^ was calculated as transition state energy. No significant difference was found in both the absorption and emission spectra of **[10,10]_2_**, indicating that the dimerization has little influence on the frontier orbitals of [10]CPP.

In a different approach, Jasti and coworkers used transition metals to form coordination complexes with 2,2’‑bipyridine‐embedded [8]CPPs as ligands.^[^ ^52^ ^]^ While they used carbazole to form a Ru(II) complex with one nanohoop, they also achieved the synthesis of a double nanohoop with a Pd(II) center as the linking unit, utilizing dimerization Pathway 4 (Scheme 1) with PdCl_2_ as the linker and with the use of AgBF_4_. Single crystals of the resulting ***N*‑[8,8]Pd** revealed the presence of two dimers with slightly different angles and significantly different packing in the solid state. Nevertheless, both dimers adopted the *trans* conformation (as shown in Figure 4), indicating a strong preference of the Pd complex for this distorted square‐planar geometry. In addition, the presence of only three resonances for the bipyridine center in the ^1^H NMR spectrum suggested high symmetry for ***N***‑**[8,8]Pd**.

**Figure 5:**
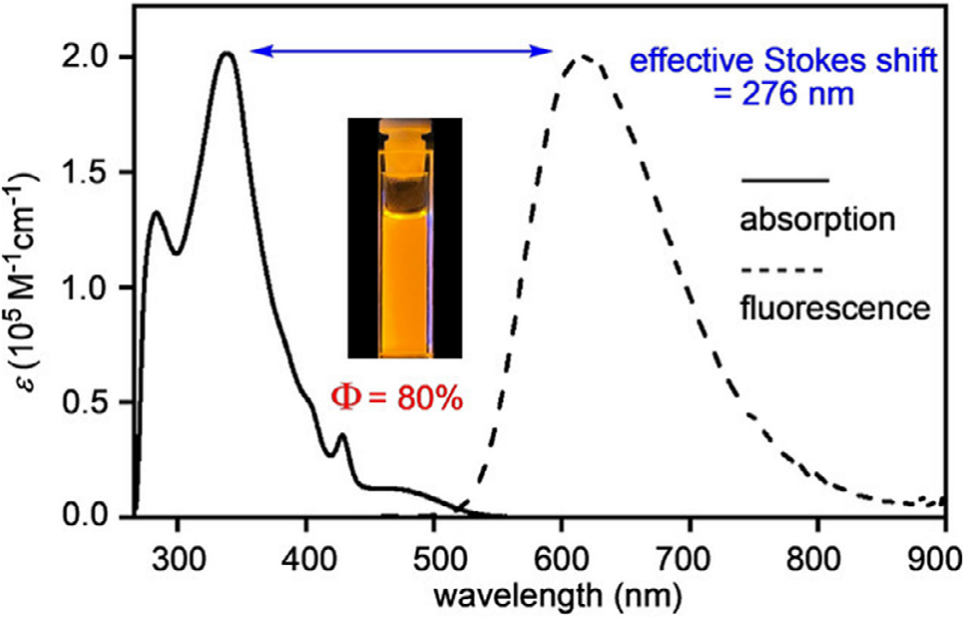
UV/vis absorption and fluorescence (excited at 410 nm) spectra of **[10,10]PAz‐THS** in CH_2_Cl_2_. Adapted from ref. [48] with permission from John Wiley & Sons – Books, 2021 Wiley‐VCH GmbH; permission conveyed through Copyright Clearance Center, Inc.

Three derivatives of a [10]CPP double nanohoop with a rigid aromatic linker were synthesized by the group of Sun.^[^ ^48^ ^]^ The synthesis was achieved by double condensation reactions of two [10]CPP diamines (Pathway 4, Scheme 1) with substituted pyrene‐4,5,9,10‐tetraone, giving compounds with a pyrazine elongated pyrene linker (PAz), namely **[10,10]PAz‐*t*‐Bu**, **[10,10]PAz‐TIPS** and **[10,10]PAz‐THS** with different degrees of solubility (Figure 4). The diamine itself was introduced through a benzo‐2,1,3‐thiadiazole unit, which was then reduced with LiAlH_4_. Interestingly, these reducing conditions led to deprotection of the TESO‐groups, as well as reductive aromatization of the hoop in parallel. As a consequence, the following condensation with the pyrenetetraone linker was performed using the already aromatized hoop, deviating from Pathway 1 in Scheme 1. Various NMR‐spectroscopic analyses were employed to confirm the structures and to investigate the cis‐ and trans‐conformers of **[10,10]PAz‐THS**. At room temperature, a fast flipping of the conformers was observed. Based on the rate constant estimated at the coalescence temperature (203 K), an energy barrier of 10.1 kcal mol^−1^ was determined. This result was confirmed by DFT calculations on an unsubstituted model compound. Contrary to the double nanohoops presented above, here the trans‐conformer was found to be lower in energy (2.3 kcal mol^−1^) than the *cis* form. **[10,10]PAz‐THS** was further investigated by optical spectroscopy in various solvents and showed solvatofluorochromic behavior. The highest fluorescence quantum yield of 80% was determined in CH_2_Cl_2_, with an orange emission at 616 nm (Figure 5). The dominant absorption at 340 nm corresponded to the [10]CPP hoop. Finally, the host–guest behavior of **[10,10]PAz‐THS** with C_60_ was investigated *via* UV/vis spectroscopic titration, and the formation of a 1:2 host–guest complex with negative cooperativity and binding constants of *K*_1_  =  4.2·10^5^ m ^−1^ and *K*_2_  =  3.6·10^3^ m ^−1^ was observed.

Subsequent work of Sun and coworkers focused on achieving control over the flipping through configurational stability.^[^ ^53^ ^]^ They designed two new types of double nanohoops utilizing the condensation approach established before. Therefore, they incorporated a pyrene‐dione unit with long alkyl chains into a nanohoop using a **[9]PhP** precursor. The alkyl chains were installed to inhibit the flipping motion. The resulting hoop was aromatized, then submitted to a condensation reaction with the above‐mentioned [10]CPP diamine, resulting in **[10,11]Paz** (Figure 4). A double condensation of two pyrene dione‐containing hoops with benzene‐1,2,4,5‐tetramine gave **[11,11]PAz** with the longest linker in the series (Pathway 4, Scheme 1). As expected, the long chains functioned as steric blockers, making the flipping impossible in **[11,11]PAz** and only partially possible for the [10]CPP in **[10,11]PAz**. This was confirmed by VT‐NMR spectroscopic and DFT studies. The conformational restrictions were reflected in the optical properties of both compounds. While the emission of **[10,11]PAz** followed Kasha's rule, solvatofluorochromic behavior was also observed. **[11,11]PAz** displayed solvent‐dependent anti‐Kasha behavior with dual fluorescence as evidenced by emission spectra and fluorescence lifetime measurements. Calculations of natural transition orbitals indicated CT from the CPP (donor, D) to the linker (acceptor, A). The two emission bands at 490 nm and 590 nm in **[11,11]PAz** were thus assigned to the locally excited and the CT state, respectively. The authors suggested that the high symmetry of **[11,11]PAz** (D‐A‐D) could account for a slow internal conversion, leading to dual fluorescence in **[11,11]PAz** but not in **[10,11]PAz** (D‐A‐D’). UV/vis titration experiments revealed the formation of a 1:2 host–guest complex of **[11,11]PAz** with C_70_. Although the cooperativity was negative with *K*_1_  =  2.3·10^5^ m ^−1^ and *K*_2_  =  1.4·10^3^ m ^−1^, the binding constant *K*_1_ was much higher compared to that of [11]CPP (*K*_1_  =  6.7·10^3^ m ^−1^). This was explained with the larger π‐surface of the pyrene‐containing nanohoop.

In 2023 Fang et al. reported on the synthesis of the two double nanohoops **[9,10]TP** and **[10,10]TP** that showed unusual supramolecular interactions.^[^ ^49^ ^]^ The synthesis was achieved in a two‐step macrocyclization strategy (Pathway 3 in Scheme 1), starting from a triphenylene derivative. The two paraphenylene loops were generated in a consecutive fashion: first, precursors of different sizes (**[7]PhP** and **[8]PhP**) were introduced to form the lower ring (Figure 4, bottom). After aromatization, a second precursor (**[7]PhP**) was attached, forming the upper loop. The structures were confirmed by mass spectrometry and NMR spectroscopy, supplemented by X‐ray diffraction of fullerene complexes and DFT calculations. While the absorption properties of both double hoop structures followed the same trend, the fluorescence spectra resembled only the smallest hoop size. For **[9,10]TP** the fluorescence spectrum was nearly identical to that of [9]CPP, while the normalized spectrum of **[10,10]TP** aligned with that of [10]CPP. The estimated *Φ*_F_ values were lower than the ones determined for the respective [*n*]CPPs.^[^ ^19^ ^]^ By slow crystallization in the presence of excess fullerenes C_60_ or C_70_, 2:1 host–guest complexes with **[9,10]TP** could be isolated, in which the upper rings of two double hoops shared a fullerene molecule. For **[10,10]TP** an unusual 2:3 host–guest complex was observed in the solid state, where the lower rings of **[10,10]TP** were found to complex another two C_60_ in addition to the shared fullerene. In solution, the formation of a 1:1 complex of **[10,10]TP** and C_60_ was indicated by MALDI‐TOF mass spectrometry, and a binding constant of approximately 4.9 × 10^5^ m ^−1^ was determined.

### 1.5 Lemniscular Double Nanohoops

**Figure 6:**
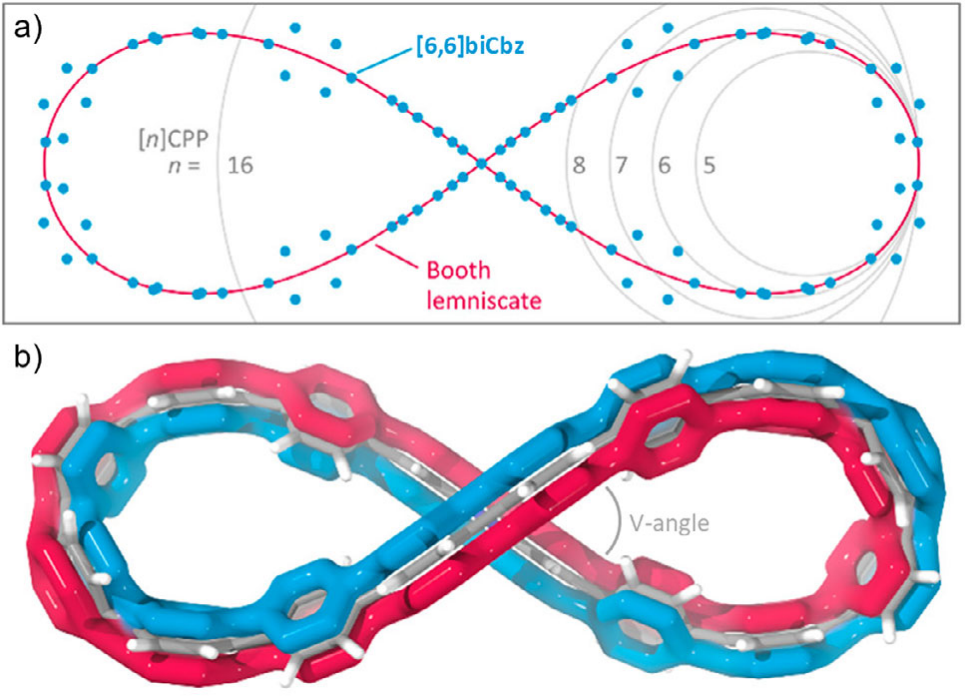
Representation of lemniscular double nanohoops based on the structure of **(*P*)‐[6,6]biCbz** determined by DFT calculations (ωB97XD/6‐31G(d,p)). a) **[6,6]biCbz** fitted with a Booth lemniscate; [*n*]CPPs of different sizes are projected for comparison. b) Illustration of the interlocked π‐system in **[6,6]biCbz**, demonstrated as red and blue surfaces. Adapted from ref. [57] with permission from American Chemical Society. Copyright 2019 American Chemical Society.

Figure‐of‐eight‐shaped double nanohoops will be discussed in the fourth class. When projected onto a plane, these double nanohoops can be fitted to the model of a Booth lemniscate, which explains the naming of the category (Figure 6a). Based on the geometry of the linkers, the macrocycles formed are interlocked in the center, allowing for axially chiral structures. The resulting lemniscates are defined, among other things, by their linking number *Lk* and differ in the dihedral angle between the planes of the central linker, hereafter named the “V‐angle” (Figure 6b). The π‐conjugation of such twisted macrocyclic aromatic structures can (1) adapt planar or radial orientation (of *p*_z_‐orbitals) and (2) be Hückel‐like (for even *Lk*) or Möbius‐like (for odd *Lk*).^[^ ^54^, ^55^, ^56^ ^]^ In this section, we give an overview of double nanohoops that adapt a figure‐of‐eight geometry with radial conjugation.

**Figure 7:**
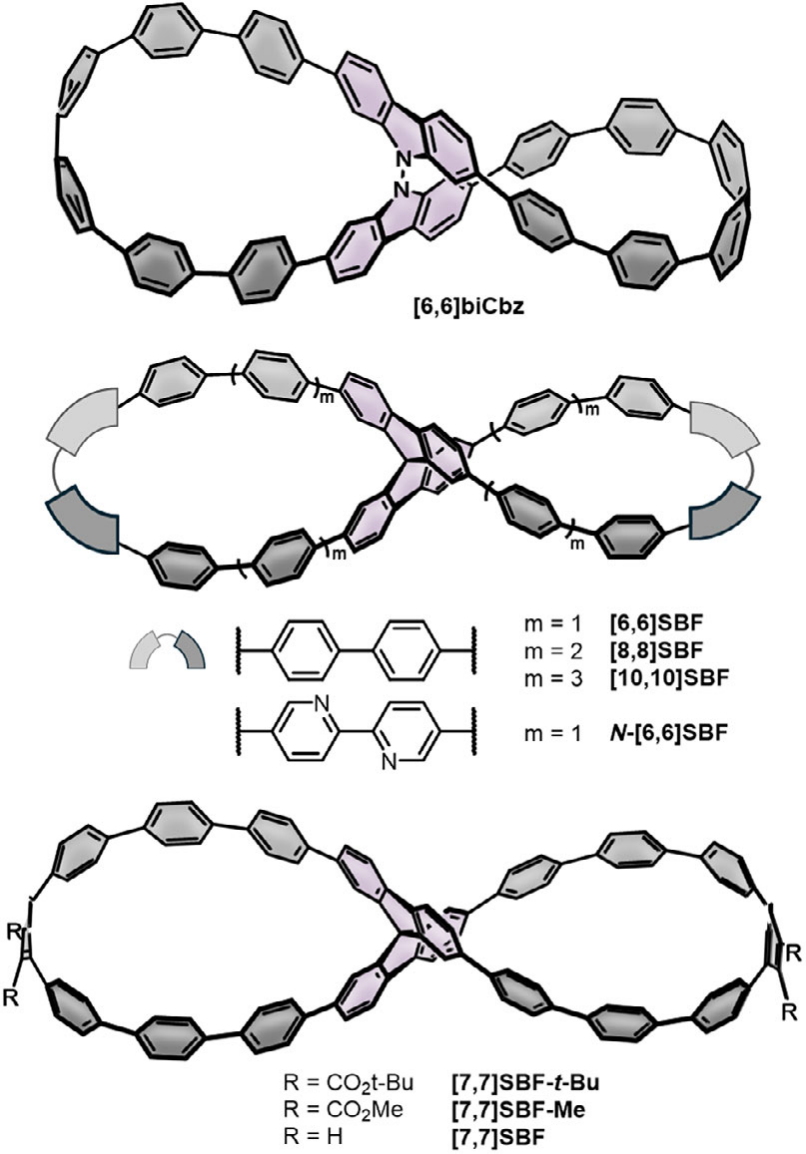
Lemniscular double nanohoops **[6,6]biCbz, [6,6]SBF, [8,8]SBF, [10,10]SBF, *N*‐[6,6]SBF, [7,7]SBF‐*t‐*Bu, [7,7]SBF‐Me**, and **[7,7]SBF**.

This type of conjugation was first obtained in 2019 by the Stępień group using an *N*,*N*’‐bicarbazole as a central unit (**[6,6]biCbz** in Figure 7).^[^ ^57^ ^]^ In the synthesized lemniscular structure, the π‐surface is half‐twisted twice, giving rise to a Hückel‐type π‐conjugation with *Lk*  =  2. At the same time, the π‐system is decoupled along the N─N bond, resulting in a lemniscate with 16 Clar sextets. The Suzuki‐Miyaura cross‐coupling of borylated bicarbazol with **[3]PhP** as kinked terphenylene precursor (Pathway 2 in Scheme 1) and subsequent Yamamoto homocoupling to form the hoop were performed in a microwave reactor. An X‐ray single crystal structure of the non‐aromatized precursor confirmed the lemniscular conformation.

Compound **[6,6]biCbz** was investigated *via* NMR spectroscopy, mass spectrometry and solid‐state Raman spectroscopy, the latter showing similarities to [6]‐ and [7]CPP. DFT calculations of the structure gave further information about the mean CCCC torsional angles between the phenylene units (between 29.2° and 39.2°) as well as bend angles (ranging from 3.3° to 12.2°) within phenylene units, both lying within the range of [*n*]CPPs.^[^ ^37^, ^58^ ^]^ A V‐angle of 70.9° was determined for the bicarbazole unit. Δ*E*_HOMO‐LUMO_ resulted in a smaller value of 3.35 eV for **[6,6]biCbz** compared to 3.66 eV for [16]CPP, attributed to the higher curvature in the former. Two methods were employed to estimate the hoop strain energies, resulting in values of 122.5 kcal mol^−1^ (from pseudo‐radial breathing mode frequency method) and 102.7 kcal mol^−1^ (from homodesmotic calculations). The absorption maximum of **[6,6]biCbz** at 357 nm was bathochromically shifted compared to [16]CPP (339 nm), and the shift of the emission maximum to 496 nm (415 nm and 438 nm for [16]CPP) was even greater.^[^ ^58^ ^]^ This shift, together with the *Φ*_F_ of 36% matches the fluorescence properties of the smaller [9]CPP.^[^ ^19^ ^]^ The two atropisomers of **[6,6]biCbz** were separated by supercritical fluid chromatography on a chiral stationary phase and their chiroptical properties were examined by circular dichroism (CD) and CPL spectroscopy (Table 4). The lemniscate **[6,6]biCbz** was further investigated computationally in 2020, showing that by introducing both donors and acceptors, Δ*E*_HOMO‐LUMO_ decreases, and the HOMO/LUMO distributions are modulated, modifying the electronic transition properties and second‐order nonlinear optical response.^[^ ^59^ ^]^ Another computational work reported that with increasing size of the loops, the hoop strain energies decrease and the V‐angle becomes greater.^[^ ^60^ ^]^ As demonstrated in 2022, **[6,6]biCbz** was also the first purely organic system in which Raman optical activity and CPL were simultaneously detected, both emerging in the same electronic excitation.^[^ ^61^ ^]^

**Table 4:** Optoelectronic properties of lemniscular double nanohoops.^[^ ^57^, ^62^, ^63^, ^64^ ^]^

Compound	*λ*_abs,max_ (nm)^a)^	*ε* (10^4 ^ m ^−1^ cm^−1^)	*λ*_em,max_ (nm)^a)^	Φ_F_ ^a)^	Hoop strain (kcal mol^−1^)^b)^
**[6,6]biCbz**	357	14.2	496	0.36	102.7
**[6,6]SBF**	359	17.6	493	0.37	92.2
**[8,8]SBF**	355	25.5	468	0.64	73.1
**[10,10]SBF**	353	28.5	454	0.86	61.8
***N*‐[6,6]SBF**	363	20.3	–	–	85.2
**[7,7]SBF‐*t*‐Bu**	357	∼17	482 499^c)^	0.002 0.012^c)^	92.3
**[7,7]SBF‐Me**	358	∼22	489 501^c)^	0.003 0.011^c)^	85.0
**[7,7]SBF**	–	–	–	–	95.4
**[6,6]OB8C**	339	∼22	573	0.006	–

^a)^ In CH_2_Cl_2_.

^b)^ From homodesmotic reactions.

^c)^ Powder.

In 2020, Schaub and Jasti reported on a series of fused bismacrocycles containing 9,9’‑spirobifluorene as a central motif.^[^ ^62^ ^]^ The **spiro[*n*,*n*]CPPs** (*n*  =  6, 8, and 10, Figure 7), as well as a derivative containing one 2,2’‑bipyridine unit per loop (with *m*  =  6, ***N*‐[6,6]SBF**, Figure 7), were obtained following Pathway 1 in Scheme 1. The structures of **[6,6]SBF**, **[8,8]SBF**, and ***N*‐[6,6]SBF** were confirmed by single‐crystal X‐ray diffraction and revealed figure‐eight geometries. DFT calculations resulted in V‐angles ranging from 86.7° to 87.9°. The lemniscates showed a red‐shifted absorption with *λ*_max_  =  353–363 nm compared to the [*n*]CPP series with *λ*_max_  =  335–340 nm for *n* = 5–18.^[^ ^37^ ^]^ This bathochromic shift can be attributed to the increased conjugation along the lemniscular system and, in the case of ***N*‐[6,6]SBF**, to the nitrogen doping.^[^ ^65^, ^66^ ^]^

The extinction coefficients ranged from 1.76 × 10^5^ m ^−1^ cm^−1^ for the smallest lemniscate **[6,6]SBF** to 2.85 × 10^5^ m ^−1^ cm^−1^ for **[10,10]SBF**, surpassing the values reported for [*n*]CPPs.^[^ ^37^, ^58^ ^]^ With increasing size, a blue shift in the emission, along with increasing *Φ*_F_, was observed, again confirming a behavior similar to [*n*]CPPs in optical properties. Experiments on the uptake of N_2_ and vapor analytes demonstrated that a loose packing in the bulk improves porosity and cavity accessibility, without requiring the presence of supramolecular nanochannels.

The group of Tanaka achieved the enantioselective syntheses of the ester‐substituted lemniscates **[7,7]SBF‐*t*‐Bu** and **[7,7]SBF‐Me** (Figure 7).^[^ ^63^ ^]^ Using Pathway 2 in Scheme 1, a cyclization precursor with four terminal alkynes was synthesized by fourfold cross‐coupling of tetrabromo‐spirobifluorene with TMS‐protected alkyne‐extended **[3]PhP**. After deprotection, the alkynes underwent [2+2+2] cycloaddition with acetylenedicarboxylic esters, closing the loops on each side and introducing esters as functional groups. The enantioselectivity was induced through the formation of a chiral Rh(I)/(*R*)‐H_8_‐BINAP complex during the cycloaddition and yielded (*M*)‑**[7,7]SBF‐*t*‐Bu** and (*M*)‑**[7,7]SBF‐Me** after reductive aromatization in enantiomeric ratios of 75:25 and 72:28, respectively. DFT calculations were performed for both ester‐substituted lemniscates and for the unsubstituted derivative **[7,7]SBF** (Figure 7). The determined bend angles (3.9°–14.2° in **[7,7]SBF‐*t*‐Bu**, 3.7°–12.5° in **[7,7]SBF**) for freely rotating phenylene rings were in the same range as for the carbazole‐containing **[6,6]biCbz**. The dihedral angles in **[7,7]SBF‐*t*‐Bu** (17.8°–47.0°) were larger than in **[7,7]SBF** (14.1°–34.6°), which can be ascribed to the sterically demanding ester groups. The bifluorene with calculated V‐angles of 72.4° for **[7,7]SBF‐*t*‐Bu** and 72.8° for **[7,7]SBF** was found to effectively disconnect the top and bottom parts of the π‐system across the spiro unit. Both **[7,7]SBF‐*t*‐Bu** and **[7,7]SBF‐Me** showed similar optical properties compared with the above‐mentioned figure‐of‐eight hoops **[6,6]biCbz** and **[6,6]SBF–*N*‐[6,6]SBF** with an absorption maximum at 357 nm. Emission maxima were determined in powder and solution for **[7,7]SBF‐*t*‐Bu** and **[7,7]SBF‐Me** (Table 4), and a weak AIE enhancement was observed in the solid state. The *Φ*_F_ values were significantly smaller than for **[6,6]biCbz** and **[6,6]SBF–*N*‐[6,6]SBF**, with values between 0.002 and 0.012 in both solution and powder. These values were partly ascribed to the phthalate moieties, as previously observed for ester‐substituted [*n*]CPPs.^[^ ^67^, ^68^ ^]^ Although the enantiomers of the axially chiral lemniscates were not separated, the chiroptical properties of the known mixtures were investigated and corrected by 100% ee, giving dissymmetry factors of about 1 × 10^−3^–2 × 10^−3^ for (*M*)‑**[7,7]SBF‐*t*‐Bu** and (*M*)‑**[7,7]SBF‐Me**. The absolute configuration of the major isomer was verified by comparison of the calculated and experimental CD spectra.

**Figure 8:**
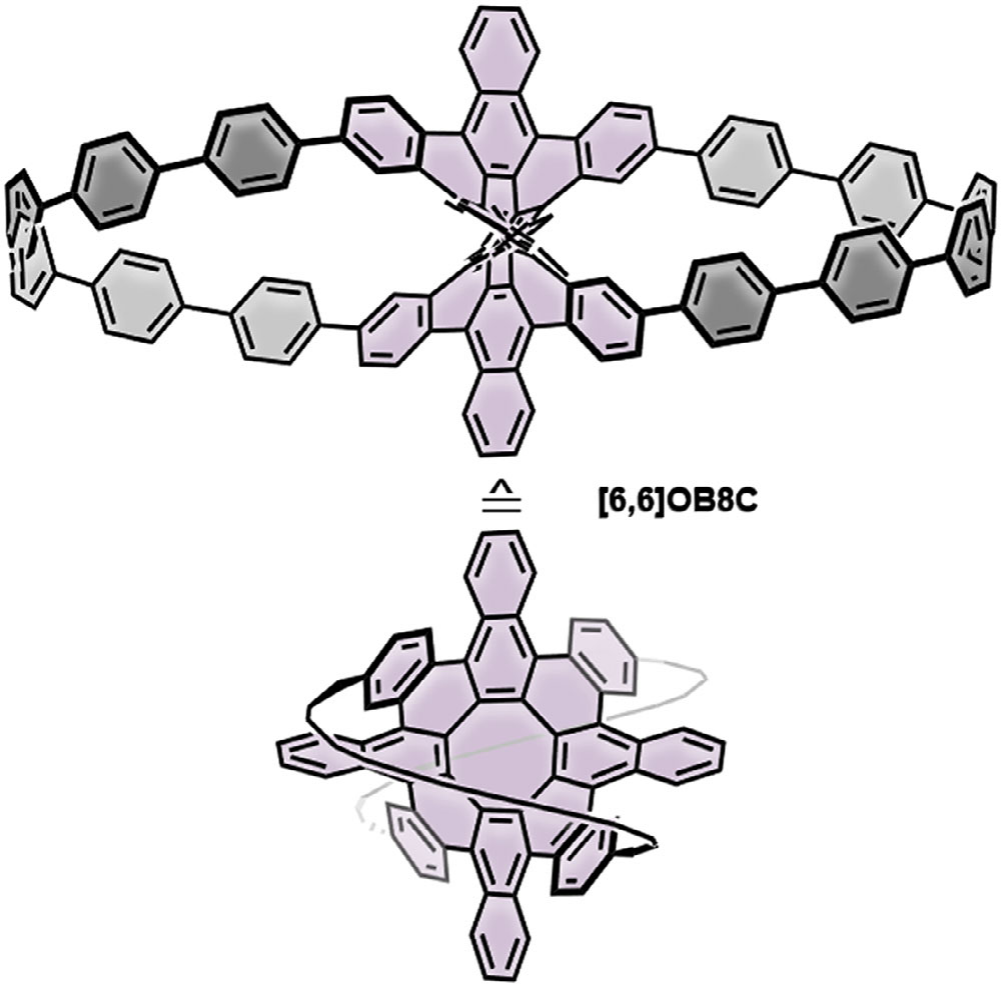
Structure of octabenzo[8]circulene‐based double nanohoop **[6,6]OB8C** shown from two perspectives. Top: Complete molecular structure with lemniscular arrangement. Bottom: Simplified schematic with the loops simplified to highlight the central linking unit.

In 2023 Miao and coworkers reported the synthesis of double nanohoops incorporating an octabenzo[**8**]circulene‐linking unit (**[6,6]OB8C**, Figure 8).^[^ ^64^ ^]^ The molecules were accessible *via* Pathway 2 (Scheme 1), using a fourfold brominated OB8C derivative and **[3]PhP**. Out of three possible ring‐closing patterns, two constitutional isomers were successfully isolated. One of these isomers was unambiguously identified by single‐crystal X‐ray diffraction and adopts a distinctive lemniscular topology, in which the central linking unit is highly twisted. The second isomer could not be fully assigned, as the precise pair of adjacent phenyl linkers forming the closure remained ambiguous with characterization limited to ^1^H NMR and mass spectrometry. Hence, only the lemniscular isomer is shown here. Their inherent chirality allowed enantiomeric resolution by chiral HPLC, followed by optoelectronic characterization. Overall, these results mark an important step toward the bottom‐up synthesis of carbon schwartzites.

### 1.6 Other Double Nanohoops

**Figure 9:**
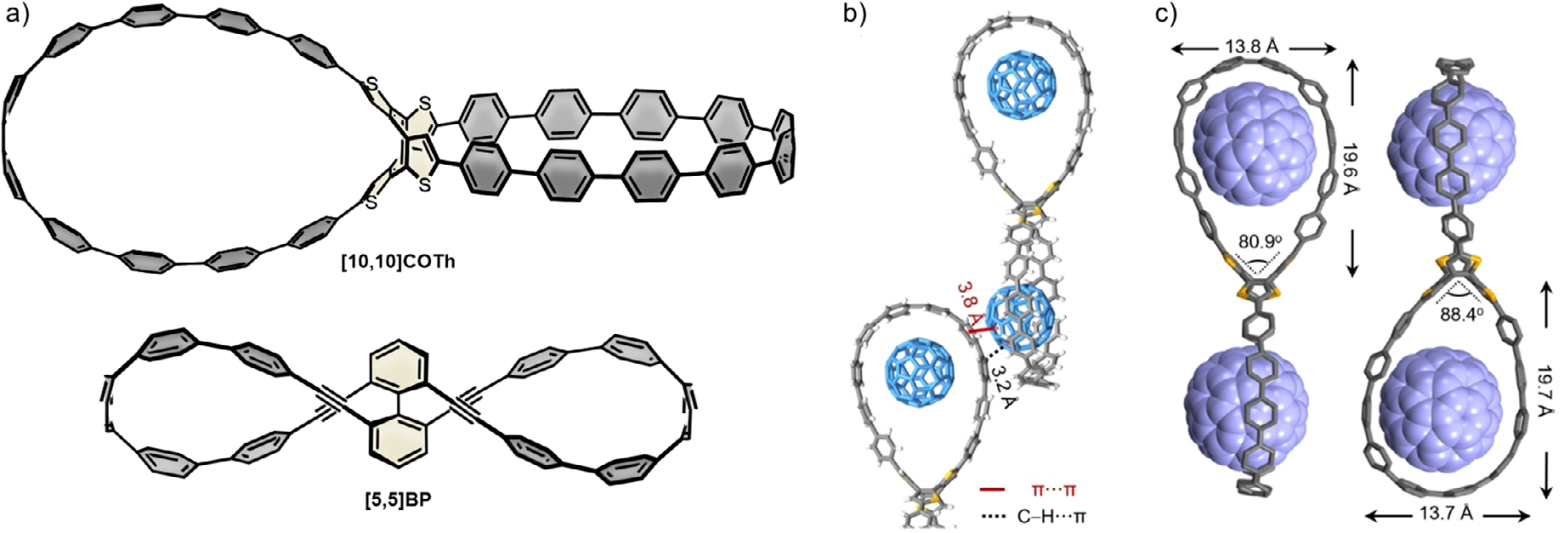
a) Structures of **[10,10]COTh** and **[5,5]BP**. b) Crystal structure of the 1:2 host–guest complex of C_60_ and **[10,10]COTh**, showing π···π and CH···π interactions. c) Crystal structure of the 2:1 host–guest complex of C_70_ and **[10,10]COTh** with V‐angles and dimensions highlighted. Hydrogen atoms and solvent molecules omitted for clarity. (b) and (c) adapted from ref [45] with permission of John Wiley & Sons – Books, 2021 Wiley‐VCH GmbH; permission conveyed through Copyright Clearance Center, Inc.

In this section we are discussing double nanohoops that cannot be categorized in the above‐mentioned types of structures. A conjugated double nanohoop by Zhan et al. was synthesized from tetrabrominated cyclooctatetrathiophene (COTh) and an unsymmetric CPP precursor **[3]PhP** (Pathway 2 in Scheme 1).^[^ ^45^ ^]^ In the resulting **[10,10]COTh**, the ellipsoid‐shaped loops are perpendicular to each other, owing to the saddle‐shaped COTh center (Figure 9a). The emission maximum of **[10,10]COTh** was observed at 520 nm with a Stokes shift of 178 nm and a *Φ*_F_ of 0.03 (Table 5). The core unit of the double nanohoop allows for a flexible cavity shape, as shown by the complexation of up to two C_60_ and C_70_ molecules. Fluorescence titrations showed a preference for C_60_ as guest, and the highest binding constant was determined for the 1:1 host‐guest complex with C_60_ (1.3 × 10^4^ m ^−1^). The 1:2 host–guest complexes were crystallized and revealed not only π···π‐interactions between the fullerene and the cavity, but also CH···π‐interactions with adjacent host molecules (Figure 9b,c). **[10,10]COTh** and its fullerene complexes were thoroughly investigated by DFT calculations, which showed that the complexation of each fullerene is thermodynamically favored by 21–27 kcal mol^−1^, as concluded from Gibbs free energy calculations. In a computational study of 2022, Stasyuk et al. analyzed the structural, electronic, and photoinduced electron‐transfer properties of the 1:1 and 1:2 host–guest complexes of **[10,10]COTh** with C_60_.^[^ ^69^ ^]^ They found that charge transfer from oligo(paraphenylene) fragments to C_60_ is energetically favorable, occurring on a sub‐nanosecond time scale.

**Table 5:** Optoelectronic properties of (not categorized) double nanohoops.^[^ ^45^, ^70^, ^71^, ^72^, ^73^ ^]^

Compound	*λ*_abs,max_ (nm)^a)^	*ε* (10^4^ m ^−1^ cm^−1^)	*λ*_em,max_ (nm)^a)^	Φ_F_ ^a)^	Hoop strain (kcal mol^−1^)^b)^
**[10,10]COTh**	342	17	520	0.03	66.4
**[5,5]BP**	355	–	515	0.16	120.6
**[6,6]‐*po*‐PCP**	334, 361	25, 24	495	0.33	–
**[6,6]‐*pm*‐PCP**	338	27	484	0.51	–
**[8,8]PA**	332	–	515	0.30	–
**[6,6]CAL**	330	14	480	0.63	–

^a)^ in CH_2_Cl_2_.

^b)^ From homodesmotic reactions.

In the figure‐of‐eight structure **[5,5]BP**, reported by the group of Tanaka in 2020, biphenyl with four ortho‐alkyne substituents acts as the chirality‐inducing linking unit (Figure 9a).^[^ ^70^ ^]^ Its Sonogashira cross‐coupling reaction with kinked oligo(paraphenylene) precursor **[5]PhP**, followed by reductive aromatization, afforded **[5,5]BP**, according to Pathway 1 in Scheme 1. Although no single crystals of **[5,5]BP** were obtained, the connectivity in the molecule was confirmed by X‐ray diffraction of its non‐aromatized precursor. As indicated by DFT calculations, the structure of **[5,5]BP** is highly twisted with a V‑angle of 96.4°. In the droplet‐shaped loops, torsional angles of 18.8°–33.7° between phenylene units were determined, and the bend angles of phenylene rings (6.5°–14.7°) were in the range of [*n*]CPPs. The absorption and emission bands were redshifted compared to a reference compound containing only one loop. This red shift was ascribed to the extended conjugation through the biphenyl unit by the ortho‐linkage. A dual emission was observed in both **[5,5]BP** and the reference compound and was related to the contributions of the acetylene moieties and the symmetry‐broken structures.^[^ ^38^, ^74^, ^75^ ^]^ As in **[6,6]biCbz**, the HOMO → LUMO transition is weakly allowed (*f*  =  0.03), according to TD‐DFT calculations, and a very strong transition with *f*  =  4.26 is observed to a higher excited state (S_5_, HOMO–1 → LUMO+2), which was attributed to the twist in the molecule. **[5,5]BP** is axially chiral, and its atropisomers were separated using HPLC on a chiral stationary phase. The chiroptical properties were investigated using CD and CPL spectroscopy, where values of Δ*ε*  =  500 m ^−1^ cm^−1^ and *g*_abs_  =  3.95 × 10^−3^ were observed, more than twice the values of the mono‐loop reference mentioned above. The absolute configurations of the atropisomers were assigned by comparison with calculated CD spectra.

**Figure 10:**
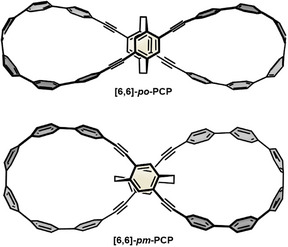
Structures of [2.2]paracyclophane‐based double nanohoops **[6,6]*po*‐PCP** and **[6,6]‐*pm*‐PCP**.

In 2023 the group of Jiang presented two lemniscular molecules with a planar chiral [2.2]paracyclophane (PCP) as the central motif (Figure 10).^[^ ^71^ ^]^ While **[6,6]‐*po*‐PCP** was synthesized according to Pathway 2 in Scheme 1, the two loops in **[6,6]‐*pm*‐PCP** were introduced successively, making use of orthogonal protecting groups. The structure of **[6,6]‐*pm*‐PCP** was confirmed by X‐ray diffraction on single crystals. NMR spectroscopy of **[6,6]‐*po*‐PCP** and **[6,6]‐*pm*‐PCP** revealed a significant shift of the signals associated with the ethylene bridges in the central PCP unit, originating from the different orientation of the cyclophane in **[6,6]‐*po*‐PCP** *vs*. **[6,6]‐*pm*‐PCP**. Topological analysis demonstrated that only **[6,6]‐*pm*‐PCP** is topologically chiral, whereas both lemniscates show chemical chirality.

In CD and CPL spectroscopic experiments, **[6,6]‐*po*‐PCP** and **[6,6]‐*pm*‐PCP** with a cyclophane core of the same chiral descriptor showed opposite chiral signals, again attributed to the orientation in the loops. Determination of the dissymmetry factors *g*_abs_ resulted in similar values (3.1 × 10^−3^ for **[6,6]‐*po*‐PCP** and 2.9 × 10^−3^ for **[6,6]‐*pm*‐PCP**), however, **[6,6]‐*pm*‐PCP** showed a larger *g*_lum_ value compared to **[6,6]‐*po*‐PCP** (3.2 × 10^−3^ and 1.8 × 10^−3^, respectively). Finally, the photophysical properties of both lemniscates were investigated and supported by DFT calculations (Table 5).

**Figure 11:**
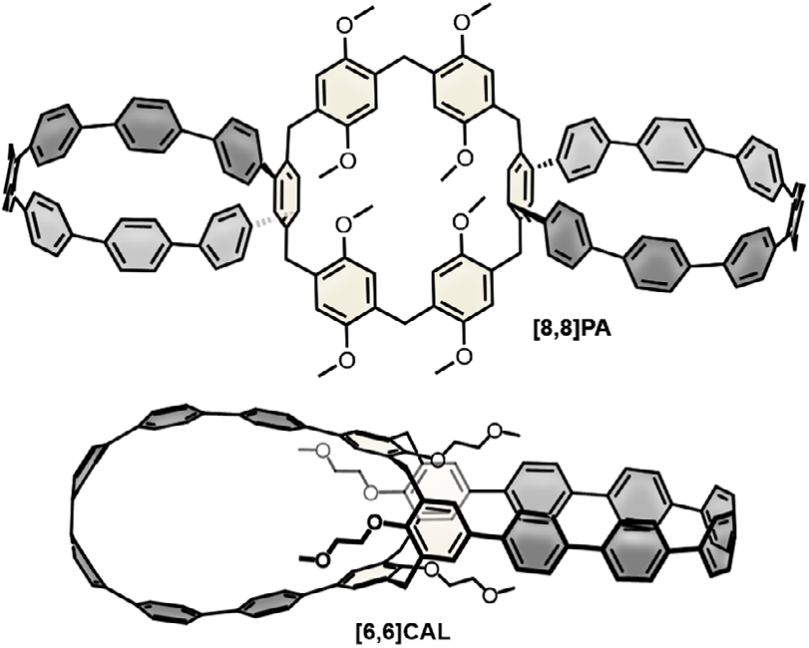
Structure*s* of chiral (*M*)‐**[8,8]PA** and of calixarene‐linked **[6,6]CAL**.

In early 2025 the group of Cheng published two diastereomers of pillar[6]arene‐based double nanohoops **[8,8]PA** (Figure 11).^[^ ^73^ ^]^ The chiral dl‐ and meso‐isomers were obtained in comparable yields (12% and 11%, respectively) *via* Pathway 1 (Scheme 1), employing **[7]PhP** as precursor. The resulting structure can be described as a trimacrocyclic system; however, due to the role of the pillar[6]arene unit as a linking element between two [8]CPP segments, we decided to include **[8,8]PA** in this review. The optical properties of **[8,8]PA** are comparable to those of [8]CPP, exhibiting a slight hypsochromic shift in both absorption and emission spectra. Notably, the *Φ*_F_ increased from 10% (for [8]CPP) to 30%. The enantiomers were not separated, and as such, CD and CPL measurements were not conducted. The diastereomers, however, were separatable by column chromatography and were even crystallized separately.

Cong and coworkers used two CPP loops to stabilize the conformation of calix[4]arene (**CAL**).^[^ ^72^ ^]^ The synthesis followed Pathway 2 in Scheme 1 and yielded **[6,6]CAL** with perpendicular orientation of the paraphenylene loops and calix[4]arene as the linker (Figure 11). Single crystals revealed an *S*_4_ symmetry and a drop shape of the loops, in which the outer phenylene rings showed the greatest degree of bending. The photophysical properties of **[6,6]CAL**, although compared to [8]CPP due to the same number of phenylene rings, were more comparable to [10]CPP. The ion recognition of the CAL unit in **[6,6]CAL** was tested in fluorescence titration experiments for various cations. Effective fluorescence quenching was observed for K^+^ and UO_2_^2+^ with binding constants of *K*  =  2.7·10^6^ m ^−1^ and *K*  =  1.4·10^6^ m ^−1^ for the 1:1 complexes, respectively. DFT calculations revealed the preferred positions of the cations within (for UO_2_^2+^) or close to the CAL cavity (for K^+^). The authors suggested that fluorescence quenching could be promoted by nonradiative processes caused by an oscillating ion movement between the two cavity sides.

A structure similar to **[8,8]PA** and **[6,6]CAL** was recently published by Hu et al. with two [8]CPPs attached to two sides of a crown ether.^[^ ^76^ ^]^ In this hybrid, however, the crown ether is conformationally very flexible, and the structure does not profit from conjugation or configurational stabilization by the linker. Therefore, this hybrid is not discussed in detail in this review.

### 1.7 Conclusions and Outlook

Synthetic advances in the past 17 years have paved the way for organic chemists to access complex molecular architectures containing several conjugated nanohoops in their structure. We herein discussed all such reported structures containing two nanohoops covalently linked by a central linking unit, so‐called double nanohoops, based on oligo(paraphenylene) loops. Their synthesis typically follows one out of four pathways, making use of kinked precursors to oligo(paraphenylenes). In each case, however, the synthesis of these exotic structures faces individual challenges, and its success always deserves appropriate acknowledgment. The optoelectronic properties of the double nanohoops discussed herein are often reminiscent of those of [*n*]cycloparaphenylenes. However, an additional benefit is their usually chiral structure, induced by the unique doublehoop geometry, which can furnish attractive chiroptical properties. With two cavities the double nanohoops can show rich host–guest‐chemistry, in particular with fullerene guest molecules. This review shows that regarding the synthesis of complex nanohoop‐based structures, the limits are creativity and endurance, which are rewarded with unique structural and attractive chiroptical as well as supramolecular properties.

## Conflict of Interests

The authors declare no conflict of interest.

## Data Availability

Data sharing is not applicable to this article as no new data were created or analyzed in this study.

## References

[1] M. A. Majewski , M. Stępień , Angew. Chem. Int. Ed. 2019, 58, 86–116, 10.1002/anie.201807004. 30006951

[2] R. Jasti , J. Bhattacharjee , J. B. Neaton , C. R. Bertozzi , J. Am. Chem. Soc. 2008, 130, 17646–17647, 10.1021/ja807126u. 19055403 PMC2709987

[3] E. S. Hirst , R. Jasti , J. Org. Chem. 2012, 77, 10473–10478, , 10.1021/jo302186h. 23126565

[4] D. Wu , W. Cheng , X. Ban , J. Xia , Asian J. Org. Chem. 2018, 7, 2161–2181, 10.1002/ajoc.201800397.

[5] M. Hermann , D. Wassy , B. Esser , Angew. Chem. Int. Ed. 2021, 60, 15743–15766, 10.1002/anie.202007024. PMC9542246 32902109

[6] R. Roy , C. Brouillac , E. Jacques , C. Quinton , C. Poriel , Angew. Chem. Int. Ed. 2024, 63, e202402608, 10.1002/anie.202402608. 38744668

[7] E. J. Leonhardt , R. Jasti , Nat. Rev. Chem. 2019, 3, 672–686, 10.1038/s41570-019-0140-0.

[8] R. Zhang , D. An , J. Zhu , X. Lu , Y. Liu , Adv. Funct. Mater. 2023, 33, 2305249, 10.1002/adfm.202305249.

[9] Y. Xu , M. von Delius , Angew. Chem. Int. Ed. 2020, 59, 559–573, 10.1002/anie.201906069. 31190449

[10] X. Chang , Y. Xu , M. von Delius , Chem. Soc. Rev. 2024, 53, 47‐83, 10.1039/D2CS00937D. 37853792 PMC10759306

[11] Z. Xu , X. Liu , C. Ge , X. Ma , Z. Sun , Org. Lett. 2025, 27, 7709–7713, 10.1021/acs.orglett.5c02467. 40626893

[12] K. Li , S. Yoshida , R. Yakushiji , X. Liu , C. Ge , Z. Xu , Y. Ni , X. Ma , J. Wu , S. Sato , Z. Sun , Chem. Sci. 2024, 15, 18832–18839, 10.1039/D4SC05849F. 39464607 PMC11506531

[13] X. Chen , Q. Deng , B. Zheng , J. Xing , H. Pan , X. Zhao , Y.‐Z. Tan , J. Am. Chem. Soc. 2024, 146, 31665–31670, 10.1021/jacs.4c10068. 39520373

[14] G. Li , L. L. Mao , J. N. Gao , X. Shi , Z. Y. Huo , J. Yang , W. Zhou , H. Li , H. B. Yang , C. H. Tung , L. Z. Wu , H. Cong , Angew. Chem. Int. Ed. 2025, 64, e202419435, 10.1002/anie.202419435. 39582429

[15] F. Alonso , M. Yus , Tetrahedron 1991, 47, 7471–7476, 10.1016/S0040-4020(01)89749-0.

[16] S. E. Lewis , Chem. Soc. Rev. 2015, 44, 2221–2304, 10.1039/C4CS00366G. 25735813

[17] P. Seitz , M. Bhosale , L. Rzesny , A. Uhlmann , J. S. Wössner , R. Wessling , B. Esser , Angew. Chem. Int. Ed. 2023, 62, e202306184, 10.1002/anie.202306184. 37606286

[18] T. J. Sisto , M. R. Golder , E. S. Hirst , R. Jasti , J. Am. Chem. Soc. 2011, 133, 15800–15802, 10.1021/ja205606p. 21913694

[19] E. R. Darzi , T. J. Sisto , R. Jasti , J. Org. Chem. 2012, 77, 6624–6628, 10.1021/jo3011667. 22804729

[20] T. Kawanishi , K. Ishida , E. Kayahara , S. Yamago , J. Org. Chem. 2020, 85, 2082–2091, 10.1021/acs.joc.9b02844. 31927928

[21] J. Xia , R. Jasti , Angew. Chem. Int. Ed. 2012, 51, 2474–2476, 10.1002/anie.201108167. 22287256

[22] P. J. Evans , L. N. Zakharov , R. Jasti , J. Photochem. Photobiol. A Chem. 2019, 382, 111878, 10.1016/j.jphotochem.2019.111878.

[23] J. Xia , J. W. Bacon , R. Jasti , Chem. Sci. 2012, 3, 3018, 10.1039/c2sc20719b.

[24] D. Wassy , M. Hermann , J. S. Wössner , L. Frédéric , G. Pieters , B. Esser , Chem. Sci. 2021, 12, 10150–10158, 10.1039/d1sc02718b. 34377404 PMC8336472

[25] Q. Huang , G. Zhuang , H. Jia , M. Qian , S. Cui , S. Yang , P. Du , Angew. Chemie 2019, 131, 6310–6315, 10.1002/ange.201900084. 30843633

[26] S. Wang , X. Li , G. Zhuang , M. Chen , P. Huang , S. Yang , P. Du , Chem. Commun. 2021, 57, 9104–9107, 10.1039/d1cc03374c. 34498619

[27] Y. Fan , S. Fan , L. Liu , S. Guo , J. He , X. Li , Z. Lian , W. Guo , X. Chen , Y. Wang , H. Jiang , Chem. Sci. 2023, 14, 11121–11130, 10.1039/D3SC04358D. 37860654 PMC10583698

[28] M. Chen , K. S. Unikela , R. Ramalakshmi , B. Li , C. Darrigan , A. Chrostowska , S. Y. Liu , Angew. Chem. Int. Ed. 2021, 60, 1556–1560, 10.1002/anie.202010556. 33021073

[29] E. R. Darzi , B. M. White , L. K. Loventhal , L. N. Zakharov , R. Jasti , J. Am. Chem. Soc. 2017, 139, 3106–3114, 10.1021/jacs.6b12658. 28151655

[30] S. Punna , D. D. Díaz , M. G. Finn , Synlett 2004, 2004, 2351–2354, 10.1055/s-2004-832845.

[31] M. Moreno‐Mañas , M. Pérez , R. Pleixats , J. Org. Chem. 1996, 61, 2346–2351, 10.1021/jo9514329. 11667001

[32] V. K. Patel , E. Kayahara , S. Yamago , Chem. Eur. J. 2015, 21, 5742–5749, 10.1002/chem.201406650. 25753916

[33] L. Jiang , Z. Peng , Y. Liang , Z. Bin Tang , K. Liang , J. Liu , Z. Liu , Angew. Chem. Int. Ed. 2023, 62, e202312238, 10.1002/anie.202312238. 37656430

[34] X. Zhang , H. Liu , G. Zhuang , S. Yang , P. Du , Nat. Commun. 2022, 13, 1–10, 10.1038/s41467-022-31281-9. 35729154 PMC9213505

[35] X. Zhang , C. Chen , W. Zhang , N. Yin , B. Yuan , G. Zhuang , X. Y. Wang , P. Du , Nat. Commun. 2024, 15, 2684, 10.1038/s41467-024-46848-x. 38538576 PMC10973529

[36] X. Zhang , H. Shi , G. Zhuang , S. Wang , J. Wang , S. Yang , X. Shao , P. Du , Angew. Chem. Int. Ed. 2021, 60, 17368–17372, 10.1002/anie.202104669. 33945657

[37] E. R. Darzi , R. Jasti , Chem. Soc. Rev. 2015, 44, 6401–6410, 10.1039/C5CS00143A. 25913289

[38] T. C. Lovell , C. E. Colwell , L. N. Zakharov , R. Jasti , Chem. Sci. 2019, 10, 3786–3790, 10.1039/C9SC00169G. 30996967 PMC6446961

[39] L. Sun , E. Kayahara , T. Nishinaga , M. Ball , D. Paley , C. Nuckolls , S. Yamago , Bull. Chem. Soc. Jpn. 2024, 97, 1–7, 10.1093/bulcsj/uoad011.

[40] K. Li , Z. Xu , J. Xu , T. Weng , X. Chen , S. Sato , J. Wu , Z. Sun , J. Am. Chem. Soc. 2021, 143, 20419–20430, 10.1021/jacs.1c10170. 34817177

[41] J. Zhao , J. Xu , H. Huang , K. Wang , D. Wu , R. Jasti , J. Xia , Angew. Chem. Int. Ed. 2024, 63, e202400941, 10.1002/anie.202400941. 38458974

[42] Y. Yang , S. Huangfu , S. Sato , M. Juríček , Org. Lett. 2021, 23, 7943‐7948, 10.1021/acs.orglett.1c02950. 34558903 PMC8524662

[43] Y. Yang , O. Blacque , S. Sato , M. Juríček , Angew. Chem. Int. Ed. 2021, 60, 13529–13535, 10.1002/anie.202101792. PMC8252656 33635576

[44] Z. A. Huang , C. Chen , X. Di Yang , X. B. Fan , W. Zhou , C. H. Tung , L. Z. Wu , H. Cong , J. Am. Chem. Soc. 2016, 138, 11144‐11147, 10.1021/jacs.6b07673. 27539737

[45] L. Zhan , C. Dai , G. Zhang , J. Zhu , S. Zhang , H. Wang , Y. Zeng , C. Tung , L. Wu , H. Cong , Angew. Chem. Int. Ed. 2022, 61, e202113334, 10.1002/anie.202113334. 34817926

[46] P. Seitz , L. Rzesny , D. Busse , X. Xiang , M. Hermann , L. Estaque , G. Pieters , B. Esser , J. Am. Chem. Soc. 2025, 147, 41610–41619, 10.1021/jacs.5c12590. 41182050 PMC12616693

[47] J. Xia , M. R. Golder , M. E. Foster , B. M. Wong , R. Jasti , J. Am. Chem. Soc. 2012, 134, 19709–19715, 10.1021/ja307373r. 23130993

[48] K. Li , Z. Xu , H. Deng , Z. Zhou , Y. Dang , Z. Sun , Angew. Chem. Int. Ed. 2021, 60, 7649–7653, 10.1002/anie.202016995. 33427356

[49] P. Fang , M. Chen , N. Yin , G. Zhuang , T. Chen , X. Zhang , P. Du , Chem. Sci. 2023, 14, 5425–5430, 10.1039/d3sc00035d. 37234903 PMC10207885

[50] Y. Ishii , S. Matsuura , Y. Segawa , K. Itami , Org. Lett. 2014, 16, 2174–2176, 10.1021/ol500643c. 24689496

[51] H. Takaba , H. Omachi , Y. Yamamoto , J. Bouffard , K. Itami , Angew. Chem. Int. Ed. 2009, 48, 6112–6116, 10.1002/anie.200902617. 19588479

[52] J. M. Van Raden , S. Louie , L. N. Zakharov , R. Jasti , J. Am. Chem. Soc. 2017, 139, 2936–2939, 10.1021/jacs.7b00359. 28212009

[53] Z. Xu , X. Liu , C. Ge , X. Tian , X. Ma , Z. Sun , J. Org. Chem. 2025, 90, 11564‐11571, 10.1021/acs.joc.5c01164. 40772614

[54] M. Stȩpień , N. Sprutta , L. Latos‐Grazyński , Angew. Chem. Int. Ed. 2011, 50, 4288–4340, 10.1002/anie.201003353. 21495122

[55] R. Herges , Chem. Rev. 2006, 106, 4820–4842, 10.1021/cr0505425. 17165676

[56] T. Tanaka , A. Osuka , Chem. Rev. 2017, 117, 2584–2640, 10.1021/acs.chemrev.6b00371. 27537063

[57] K. Senthilkumar , M. Kondratowicz , T. Lis , P. J. Chmielewski , J. Cybińska , J. L. Zafra , J. Casado , T. Vives , J. Crassous , L. Favereau , M. Stȩpień , J. Am. Chem. Soc. 2019, 141, 7421–7427, 10.1021/jacs.9b01797. 30998349

[58] Y. Segawa , A. Fukazawa , S. Matsuura , H. Omachi , S. Yamaguchi , S. Irle , K. Itami , Org. Biomol. Chem. 2012, 10, 5979, 10.1039/c2ob25199j. 22441238

[59] Y. Si , G. Yang , New J. Chem. 2020, 44, 12185–12193, 10.1039/D0NJ02637A.

[60] S. M. Bachrach , J. Org. Chem. 2020, 85, 674–681, 10.1021/acs.joc.9b02742. 31859502

[61] L. Palomo , L. Favereau , K. Senthilkumar , M. Stępień , J. Casado , F. J. Ramírez , Angew. Chem. Int. Ed. 2022, 61, e202206976, 10.1002/anie.202206976. PMC9544083 35785514

[62] T. A. Schaub , E. A. Prantl , J. Kohn , M. Bursch , C. R. Marshall , E. J. Leonhardt , T. C. Lovell , L. N. Zakharov , C. K. Brozek , S. R. Waldvogel , S. Grimme , R. Jasti , J. Am. Chem. Soc. 2020, 142, 8763–8775, 10.1021/jacs.0c01117. 32279489

[63] L. H. Wang , J. Nogami , Y. Nagashima , K. Tanaka , Org. Lett. 2023, 25, 4225–4230, 10.1021/acs.orglett.3c00895. 37219051

[64] Y. Zhang , D. Yang , S. H. Pun , H. Chen , Q. Miao , Precis. Chem. 2023, 1, 107–111, 10.1021/prechem.3c00009. 40881112 PMC12382250

[65] J. H. May , J. M. Fehr , J. C. Lorenz , L. N. Zakharov , R. Jasti , Angew. Chem. Int. Ed. 2024, 63, e202401823, 10.1002/anie.202401823. 38386798

[66] E. R. Darzi , E. S. Hirst , C. D. Weber , L. N. Zakharov , M. C. Lonergan , R. Jasti , ACS Cent. Sci. 2015, 1, 335–342, 10.1021/acscentsci.5b00269. 27162989 PMC4827663

[67] N. Hayase , Y. Miyauchi , Y. Aida , H. Sugiyama , H. Uekusa , Y. Shibata , K. Tanaka , Org. Lett. 2017, 19, 2993–2996, 10.1021/acs.orglett.7b01231. 28513181

[68] Y. Miyauchi , K. Johmoto , N. Yasuda , H. Uekusa , S. Fujii , M. Kiguchi , H. Ito , K. Itami , K. Tanaka , Chem. Eur. J. 2015, 21, 18900–18904, 10.1002/chem.201504185. 26568418

[69] O. A. Stasyuk , A. J. Stasyuk , M. Solà , A. A. Voityuk , J. Nanostructure Chem. 2024, 14, 293–306, 10.1007/s40097-022-00518-w.

[70] L. H. Wang , N. Hayase , H. Sugiyama , J. Nogami , H. Uekusa , K. Tanaka , Angew. Chem. Int. Ed. 2020, 59, 17951–17957, 10.1002/anie.202006959. 32618087

[71] J. He , M. H. Yu , Z. Lian , Y. Q. Fan , S. Z. Guo , X. N. Li , Y. Wang , W. G. Wang , Z. Y. Cheng , H. Jiang , Chem. Sci. 2023, 14, 4426–4433, 10.1039/d2sc06825g. 37123181 PMC10132256

[72] K. Xue , J.‐N. Gao , Y. Qiu , H. Wang , L.‐L. Mao , H. Cong , Org. Lett. 2025, 27, 8729–8734, 10.1021/acs.orglett.5c02677. 40707871

[73] H. Zhong , K. Lan , J. Ming , D. Zhang , C. Cheng , Org. Lett. 2025, 27, 4349–4354, 10.1021/acs.orglett.5c01097. 40221917

[74] X. Zhou , R. R. Thompson , F. R. Fronczek , S. Lee , Org. Lett. 2019, 21, 4680–4683, 10.1021/acs.orglett.9b01563. 31144823

[75] T. A. Schaub , J. T. Margraf , L. Zakharov , K. Reuter , R. Jasti , Angew. Chem. Int. Ed. 2018, 57, 16348–16353, 10.1002/anie.201808611. 30324747

[76] Y. Hu , T. Li , T. Su , W. Shi , Y. Yu , B. Li , M. H. Li , S. Zhang , Y. Q. Xu , Q. Liu , D. Wu , Y. Xu , Chem. Sci. 2025, 16, 13115–13121, 10.1039/d5sc03476k. 40556727 PMC12183571

